# Streamlined computational pipeline for genetic background characterization of genetically engineered mice based on next generation sequencing data

**DOI:** 10.1186/s12864-019-5504-9

**Published:** 2019-02-12

**Authors:** C. Farkas, F. Fuentes-Villalobos, B. Rebolledo-Jaramillo, F. Benavides, A. F. Castro, R. Pincheira

**Affiliations:** 1 Laboratorio de Transducción de Señales y Cáncer. Departamento de Bioquímica y Biología Molecular. Facultad Cs. Biológicas, Universidad de Concepción, Concepción, Chile; 2 Facultad de Medicina Clínica Alemana, Universidad del Desarrollo, Santiago, Chile; 3 Department of Epigenetics and Molecular Carcinogenesis, M.D. Anderson Cancer Center, Smithville, TX USA

**Keywords:** Sequencing, Congenic mouse, Knockout mouse, Genomic variation, Genetic interactions, Modifier genes, Genetic background, RNA-Seq variant calling, qPCR validation, *Ang*, *Cdkn1a*, *Sall2*

## Abstract

**Background:**

Genetically engineered mice (GEM) are essential tools for understanding gene function and disease modeling. Historically, gene targeting was first done in embryonic stem cells (ESCs) derived from the 129 family of inbred strains, leading to a mixed background or congenic mice when crossed with C57BL/6 mice. Depending on the number of backcrosses and breeding strategies, genomic segments from 129-derived ESCs can be introgressed into the C57BL/6 genome, establishing a unique genetic makeup that needs characterization in order to obtain valid conclusions from experiments using GEM lines. Currently, SNP genotyping is used to detect the extent of 129-derived ESC genome introgression into C57BL/6 recipients; however, it fails to detect novel/rare variants.

**Results:**

Here, we present a computational pipeline implemented in the Galaxy platform and in BASH/R script to determine genetic introgression of GEM using next generation sequencing data (NGS), such as whole genome sequencing (WGS), whole exome sequencing (WES) and RNA-Seq. The pipeline includes strategies to uncover variants linked to a targeted locus, genome-wide variant visualization, and the identification of potential modifier genes. Although these methods apply to congenic mice, they can also be used to describe variants fixed by genetic drift. As a proof of principle, we analyzed publicly available RNA-Seq data from five congenic knockout (KO) lines and our own RNA-Seq data from the *Sall2* KO line. Additionally, we performed target validation using several genetics approaches.

**Conclusions:**

We revealed the impact of the 129-derived ESC genome introgression on gene expression, predicted potential modifier genes, and identified potential phenotypic interference in KO lines. Our results demonstrate that our new approach is an effective method to determine genetic introgression of GEM.

**Electronic supplementary material:**

The online version of this article (10.1186/s12864-019-5504-9) contains supplementary material, which is available to authorized users.

## Background

The use of mouse models has resulted in a wealth of knowledge regarding gene function in animal and human diseases, including complex traits. The modern laboratory mouse is the result of careful breeding and trait selection that began in the early twentieth century [1–3]. Inbred mice, produced by brother-sister mating, are isogenic and homozygous, making it possible to know the genetic profile of the strain by typing an individual [4]. Some inbred strains have features that are valuable for transgenic [5] and embryonic stem cell (ESC) technology [6]. The 129-derived ESCs are particularly successful in germline transmission and have been extensively used in the creation of over 5000 knockout (KO) lines [6–8]. However, many ESC lines have been now derived from other strains. For example, ESCs from C57BL/6 N are used in large consortium projects (e.g., EUCOMM). After screening for an ESC clone harboring the targeted allele (e.g., KO and knockin [KI]), ESCs are typically injected into blastocysts (from a strain that differs in coat color) in order to obtain chimeras showing a mixture of black and agouti (or albino) spots, suitable to estimate the degree of chimerism. These chimeras need to be crossed with wild-type (WT) mice to test for germline transmission. The heterozygous carriers of targeted alleles are then either intercrossed, obtaining a line with mixed background, or backcrossed (typically to recipient C57BL/6), obtaining a congenic line by further backcrossing [4, 9]. However, this strategy has disadvantages; the resulting mice will contain mixed backgrounds, and the development of a full congenic line could take up to 5 years given that 10 generations of backcrosses are needed with the recipient strain [10]. Although this timeframe can be reduced when using *marker-assisted* backcrossing (*speed congenics*), it could still take at least 2.5 years [11].

An important consideration is the complex phenotypic evaluation that could result from targeted gene analysis in mixed background lines. Each individual KO or KI mouse (and the wild-type [WT] littermates) will have a different genetic background compositions, due to differences in the segregating background genes from the two parental strains [12, 13]. Thus, the different genetic backgrounds of KO/KI models could influence the resulting targeted-gene phenotype [14–18], particularly affecting the reproducibility of translational studies when mixed and/or uncharacterized backgrounds are used [19–21]. Additionally, the presence of a segment of the ESC-derived chromosome flanking the targeted gene also known as the “congenic footprint”, can confound analysis of phenotypes associated with the targeted gene [22]. The congenic footprint and its pattern of expression could lead to an inaccurate comparison between WT and KO/KI mice due to the linkage of genes at the targeted locus [23]. In line with this, several reports have shown evidence of dramatic changes in gene expression associated with flanking genes, closely related to the genetic background [22, 24–26]. These interactions could incorporate bias in dissecting the KO/KI-dependent transcriptomes, adjudicating erroneous phenotypes [23, 27–29]. Incorporation of new genome editing nuclease-dependent techniques is certainly addressing this problem, allowing the generation of GEM on any inbred strain without using ESCs or chimeras. Still, novel variants could be fixed in these lines due to off-target effects from the Cas9 model generation [30] and/or genetic drift over time [31], justifying the need for accurate genetic background characterization in every GEM line used. Although background characterization can be performed using SNP genotyping in different platforms [32], these methods test a limited number of loci, not always related to protein coding genes, and do not detect novel variants.

Next generation sequencing (NGS) enables high throughput sequencing of genes and genomes at relatively low cost. However, resulting NGS data is very complex, and additional computational methods should be available for the scientific community to characterize the genetic background of GEM lines. Here, we present a computational pipeline that uses NGS data from whole genome shotgun sequencing (WGS), whole exome sequencing (WES) and/or RNA-Seq to detect the nature, ploidy and amount of introgressed variants in GEM lines. This pipeline can generate genome-wide plots of variants per genotype, detect congenic footprints and identify potential modifier genes, which will enable a better understanding of the phenotypic outcomes in studies using partially congenic or mixed background GEM lines, as well as to unravel novel genetic interactions in these models.

## Methods

### Isolation of primary mouse embryonic fibroblasts (MEFs) and cell cultures

We obtained *Sall2* KO mice from Dr. Ryuichi Nishinakamura (Kumamoto University, Kumamoto, Japan) by a material transfer agreement (MTA, 2010). Genotyping of these mice was as previously described [33] and their housing was performed according to the Animal Ethics Committee of the Chile’s National Commission for Scientific and Technological Research (CONICYT, Protocol FONDECYT project 1,151,031). At 13,5 days *post coitum* female mice were euthanized with a CO_2_ inhalation process, and MEFs from *Sall2* WT and KO embryos were isolated as described previously [33]. Mice were routinely genotyped by isolating tail DNA as previously reported [33]. In brief, 1 μL of genomic DNA was used for PCR analysis using the following oligonucleotides: forward, 5′-CACATTTCGTGGGCTACAAG-3′; reverse, 5′-CTCAGAGCTGTTTTCCTGGG-3′; and Neo, 5′-GCGTTGGCTACCCGTGATAT-3′. The sizes of the PCR products were 188 bp for the WT and 380 bp for the KO.

### Cell culture

*Sall2*^*+/+*^, *Sall2*^*+/−*^, and *Sall2*^*−/−*^ primary and immortalized MEFs were cultured in DMEM supplemented with 10% heat inactivated fetal bovine serum (FBS, GE Healthcare HyClone), 1% glutamine (Invitrogen), and 0.5% penicillin/streptomycin (Invitrogen). Experiments with primary *Sall2*^*+/+*^ and *Sall2*^*−/−*^ MEFs were performed with early passages (passages 3–4). Immortalized *Sall2*^*+/+*^ and *Sall2*^*−/−*^ MEFs were obtained using SV40 large T antigen based on a modified protocol from Zhu et al. [34]. For transfection of primary MEFs, we used Lipofectamine 2000 (Invitrogen) and 2 μg of SV40 large T antigen expression vector (Addgene Plasmid #9053). After cell transfection, we proceeded to select for low density. To complete the immortalization process, 5–6 post-transfection passages were carried out. Human embryonic kidney epithelial cells (HEK293; American Type Culture Collection CRL-1573™) were cultured in DMEM supplemented with 10% FBS, 1% glutamine, and 0.5% penicillin/streptomycin.

### RNA-Seq analysis for the detection of differentially expressed genes (DEGs)

We purified RNA (Qiagen) from *Sall2*^*+/+*^, *Sall2*^*+/−*^ and *Sall2*^*−/−*^ MEFs treated or not with doxorubicin 1 μM (Sigma Aldrich) for 16 h. RNA-Seq libraries were prepared at the University of Cambridge sequencing facility (UK). Sequencing in a Next-seq 500 machine yielded an output of 400 gigabases and four FASTQ files per sample. We merged the FASTQ files matching each sample and aligned the reads against the mouse genome assembly (mm10 build) using the HISAT2 aligner (v2.0.5.1, default settings) [35]. We sorted the BAM files using the SortSam.jar script from Picard tools and implemented the HTSeq code (union mode) to quantify the number of reads per gene in each BAM file [36]. The GTF file (genes.gtf) used in HTSeq was from the igenomes repository (mm10, Illumina). Prior to testing for differential expression, we normalized the count table with the RUVSeq package available in Bioconductor (R, Bioconductor: https://www.bioconductor.org/packages/release/bioc/html/RUVSeq.html) with in-silico empirical negative controls and RUVg normalization [37]. The edgeRun code (exact test, y = 50,000) was used to perform differential expression analysis between WT and KO samples [38]. We selected further DEGs with an FDR < 0.001. Gene ontology analysis was performed by using the InnateDB database (https://www.innatedb.com) [39].

### Computational pipeline for variant calling and characterization from the NGS data. Galaxy platform

We uploaded individual BAM files from the RNA-Seq data to the main Galaxy platform (https://usegalaxy.org/). After sorting, genome-wide simple diploid calling was applied using Freebayes (https://github.com/ekg/freebayes). We filtered variants from the resulting raw VCF (Variant Call Format) files using the VCFlib program (https://github.com/vcflib/vcflib) with the following criteria: -f “DP > 10” (Depth over 10 reads) and -f “QUAL > 30” (minimum Phred-scaled probability of error over 30). Chromosomal histograms were plotted using an “in-house” R script (see “script outline” in https://github.com/cfarkas/Genotype-variants). For identification of common variants in KO animals not present in their WT counterparts, we used several tools from the VCFlib toolkit available in Galaxy. We started intersecting KO VCF files using the VCF-VCF intersect program (reference genome mm10) and annotated genotypes (VCF annotate genotypes) using calls from the WT file. We filtered the resulting annotated VCF file by selecting lines that did not match those of the WT (Filter and Sort). An output file with the KO-linked variants was obtained.

### Bash

Four BASH scripts were used sequentially to 1) sort bam files with SAMtools (sort_bam.sh), 2) perform variant calling with Freebayes (variant_collection.sh, parameters described above), 3) filter variants in each VCF file with VCFlib/Bcftools dependencies (filtering_combined_mouse.sh, parameters for VCFlib described above) and 4) dissect KO/KI-linked variants and visualize common variants for each genotype with R (**genotype_variants_mouse.sh, see** **https://github.com/cfarkas/Genotype-variants**).

### Visualization of variants in R

We developed a script written in R (genotype_variants.R) for proper visualization of variants across mouse chromosomes. The script takes the intersected VCF files from WT and KO mice in VCF format as inputs and produces an output of variant frequency per chromosome. The script also includes statistical detection of chromosomes with KO-linked variants in the experiments. We tested the frequency distribution of variants with the Cochran-Armitage test for trend distribution, available in the DescTools package implemented in the R statistical program (https://cran.r-project.org/web/packages/DescTools/index.html). Detected variants were binned every 10 million base pairs according to their chromosomal coordinates, ordered in a contingency table and plotted. After this, a Cochran-Armitage test for trend distribution was implemented to identify chromosomes containing KO-linked variants, based on the frequency distribution of WT and KO genotypes. Graphics were done with the ggplot2 package, implemented in R (https://cran.r-project.org/web/packages/ggplot2/index.html).

### Real-time PCR

We isolated RNA from cells using TRIzol (ThermoFisher Scientific, Inc.) followed by chloroform and isopropanol extraction. The RNA samples were treated with Turbo DNA-free Kit (Invitrogen) to eliminate any residual DNA from the preparation. Total RNA (2 μg) was reverse transcribed using the M-MLV reverse transcriptase (PROMEGA) and 0.25 μg of Anchored Oligo(dT)20 Primer (Invitrogen; 12,577–011). We performed qPCR reactions in triplicate using KAPA SYBR FAST qPCR Master Mix (2X) Kit (Kapa Biosciences) and primer concentrations of 0.4 μM (Additional file 10: Table S1). Cycling conditions were as follows: initial denaturation at 95 °C for 3 min, then 40 cycles with 95 °C for 5 s (denaturation) and 60 °C for 20 s (annealing/extension). To control specificity of the amplified product, a melting-curve analysis was carried out. No amplification of unspecific product was observed. Expression of each gene was relative to *Polr2a* gene (RNA pol II) and plotted as fold change compared to control in each case.

### Western blot analysis

Proteins from cell lysates (50–80 μg of total protein) were fractionated by SDS-PAGE and transferred for 1 h at 200 mA to PVDF membranes (Immobilon; Millipore) using a wet transfer system. The PVDF membranes were blocked for 1 h at room temperature in 5% nonfat milk in TBS-T (TBS with 0.1% Tween), and incubated with primary antibody at an appropriate dilution at 4 °C overnight in blocking buffer. After washing, the membranes were incubated with horseradish peroxidase-conjugated secondary antibodies diluted in TBS-T buffer for 1 h at room temperature. Immunolabeled proteins were visualized by ECL (General Electric Healthcare, Amersham, UK). Antibodies used for Western blotting were as follows: anti-angiogenin (1:500, ab10600; Abcam), anti-p53 (1:500, PAb240; Abcam), anti-p21 (1:500, sc-6246; Santa Cruz Biotechnology), anti-β-actin (1:10000, C4; Santa Cruz Biotechnology), and anti-SALL2 (1:1000, HPA004162; SIGMA).

### Transient transfections and viral infection

For transient transfection, 1.5 × 10^6^ immortalized MEFs (iMEFs) from *Sall2*^*+/+*^ mice were electroporated using 30 μg of plasmids at 1150 V for 30 milliseconds (NEON Transfection System, Thermo Fisher Scientific). For transduction of *Sall2* shRNA into iMEFs, lentiviral particles were packaged in HEK293 cells by co-transfecting pCMV-dR8.2 dvpr (Addgene Plasmid #8455), pCMV-VSVG (Addgene plasmid #8454) and pLKO.1 (Addgene Plasmid #8453) containing the 5’-CCGGAAGTCATGGATACAGAAGCACA**CTCGAG**TGTGCTCTGTATCCATGAC**TTTTTTT**G -3′ (loop & stop in bold) sequence, which targets exon 2 of *Sall2*. The medium was changed every 24 h with 9 μg/mL of polybrene and 24, 48 and 72-h supernatants were filtered through a 0.45 μm filter, collected and added to WT iMEF cells in each case. iMEF cells were selected with 5 μg/mL of puromycin and further recovered with fresh DMEM medium.

### CRISPR-Cas9 KO generation

WT iMEFs were electroporated as described above, with vectors encoding CRISPR-Cas9 in frame with PaprikaRFP (ATUM, DNA TWOPOINTO INC) using the following guide RNA sequences: GGTGAGCGAGGAATTCGGTC and TAGTCTAGGTGCTCCGGTAC targeting the largest exon of the mouse *Sall2* gene (exon 2). These two proteins can be efficiently produced from one coded peptide that relies on the self-cleaving 2A peptide to allow translational skipping [40]. At 16 h following electroporation, the top 2% of the brightest cells were sorted with BDFACSAria III cell sorter (BD Biosciences-US), and pools of 100 cells were plated. The pools were grown for two weeks, and Western blotting against SALL2 was performed to identify silenced cells. Genomic PCR and further sequence analysis were used to confirm CRISPR-Cas9-mediated edition of the *Sall2* locus.

## Results

### Genome-wide detection and distribution of variants from GEM lines

**Fig. 1:**
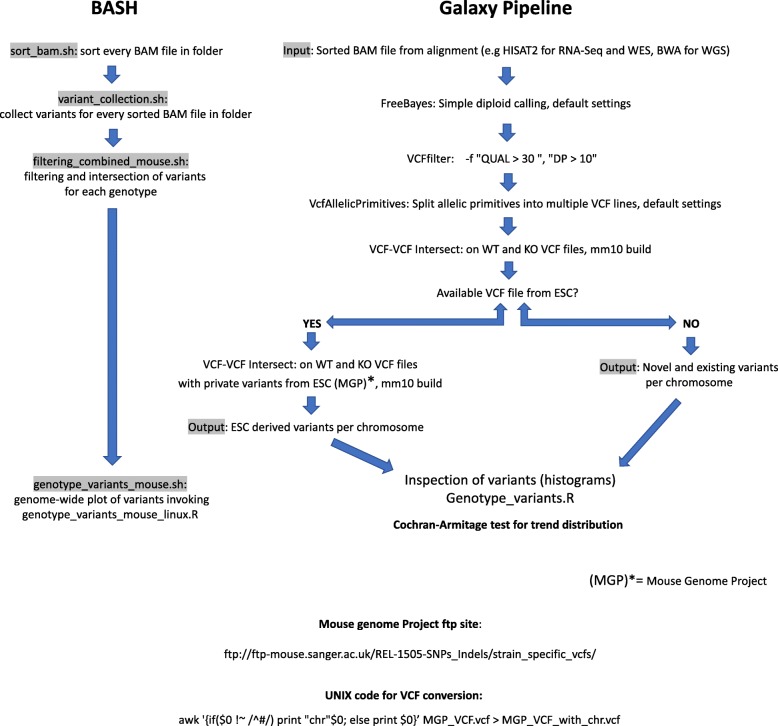
A computational pipeline for the detection of ESC-derived introgressed variants. Galaxy Platform: The pipeline starts with the input of the aligned BAM file from each genotype on the corresponding mouse genome build (e.g., HISAT2 output on the mm10 genome build for RNA-Seq data, BWA output from WES or WGS). The Freebayes variant caller program (simple variant calling) produces a VCF file from every BAM file. We filtered these VCF files using VCFlib, with the following parameters: -f “QUAL > 30”, −f “DP > 10”. Next, VCF-VCF intersect program intersects VCF files from each genotype to obtain the average variation on each genotype (mm10 build, default parameters). If the genome of the ESC used for targeting is available, and variants are correctly characterized, we can use these calls to intersect ESC introgressed variants in the VCF files from each genotype. We used VCF files available in the mouse genome project (http://www.sanger.ac.uk/science/data/mouse-genomes-project) based on the GRCm38 mouse genome release, compatible with the mm10 build (release REL-1505-SNPs_Indels). In these VCF files, the prefix “chr” in every variant call line needs to be added for compatibility with Freebayes VCF files (see UNIX code). If the genome of the ESC is not available, novel and ESC-derived variants are obtained. To confirm chromosomes with a differential distribution of variants among genotypes, we applied the Cochran-Armitage test for trend distribution. BASH: Input BAM files from RNA-Seq/WES/WGS are sorted and indexed with the sort_bam.sh script, then, variant_collection.sh script is applied for variant collection in each BAM file with Freebayes. Filtering and intersection are proceeded as described in the Galaxy platform with the filtering_combined_mouse.sh script. At this step, intersection with ESC-derived variants from the mouse project can be applied to the intersected VCF files (see Github: https://github.com/cfarkas/Genotype-variants). Finally, genome-wide plots of the intersected variants per genotype including KO-linked variants can be obtained by applying the genotype_variants_mouse.sh script

Because there are several sources of genetic variation occurring in KO mice (Additional file 1), we designed a pipeline that allows identification and genome-wide plotting of variants from NGS data, including WGS, WES, and RNA-seq. The pipeline can be implemented both in the Galaxy platform [41, 42] and directly in BASH using several scripts (See METHODS section). If the VCF file of the ESC is available, the pipeline can also identify ESC-introgressed variants (Fig. 1).

**Fig. 2:**
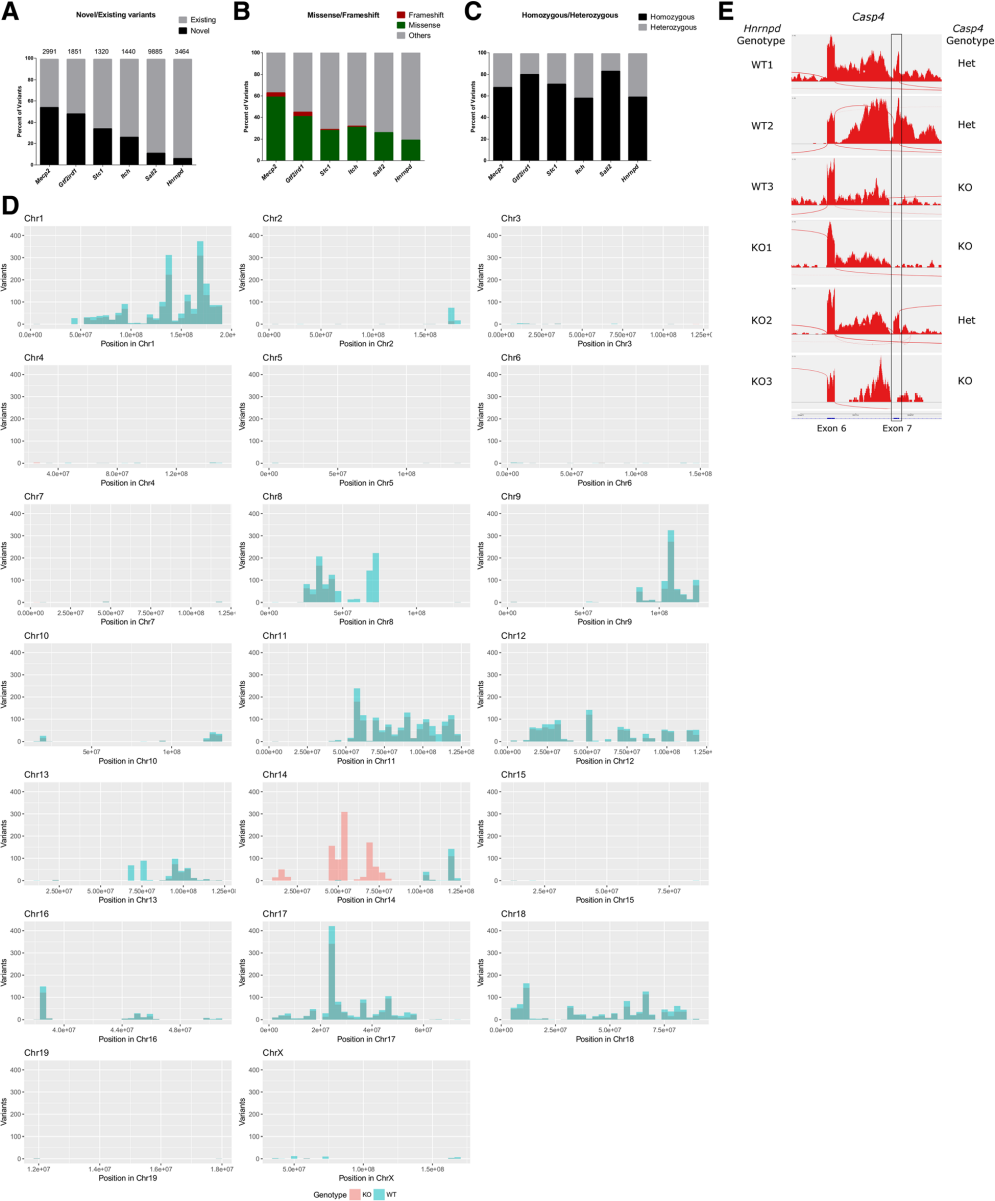
Genome-wide detection and distribution of variants from GEM mice. **a** Interleaved bar graph showing the percentage of novel (black bars) and existing (grey bars) variants characterized by the variant effect predictor (VEP) in each KO. The total number of variants is depicted above each bar. **b** Percentage of frameshift variants (red), missense variants (green) and other variants (grey) characterized in every KO. **c** The ratio between homozygous (black) and heterozygous variants (grey) expressed as percentages in every KO. **d** Histogram of 129P2OlaHsd private variants per chromosome in *Sall2* WT and null embryos. We binned the genomic coordinates of each chromosome every 10 million bases and plotted the variants of each genotype as frequency histograms according to these positions. Blue bars represent variants from one WT embryo and red bars represent the average variants from three *Sall2-*null embryos. **e** Sashimi plots from three biological replicates of WT and KO RNA sequencing samples from *Hnrnpd* KO. Per-base expression is plotted on the y-axis of Sashimi plot; genomic coordinates on the x-axis, and the gene structure are represented on the bottom (in blue, obtained from the USCS server). We obtained the genotypes of the *Casp4* gene from each replicate with Freebayes based on at least one SNP call. We highlighted the expression of exon 7 in a black rectangle to denote its absence in *Casp4* null samples

We first tested the pipeline in silico using RNA-Seq data from five congenic KO lines publicly available in GEO datasets with the following accession numbers: GSE71126, GSE81082, GSE47395, GSE65686 and GSE83555 (*Mepc2, Gtf2ird1, Stc1, Itch and Hnrnpd/AUF-1* targeted genes, respectively). In addition, we generated and analyzed our own RNA-Seq data from MEFs isolated from *Sall2* WT and *Sall2-*knockout embryos (*Sall2* KO). The *Sall2* gene targeting was done in 129P2/OlaHsd (129P2)-derived ESCs (E14.1) [43]. The pipeline was applied to call novel and existing variants from each experiment. Further characterization of the variants was done with the variant effect predictor (VEP) algorithm [44]. Focusing on KO samples, we found that the number and ratio of novel/existing variants varied among the KO lines, and that novel variants accounted for more than 50% of the total variants, as seen in *Mecp2* and *Gtf2ird1* KOs (Fig. 2a). We also observed that the number of missense and frameshift variants were positively correlated with the number of novel variants (Fig. 2b) (*P* = 0.0167, Spearman’s correlation). The ratio of homozygous/heterozygous variants among KO lines also varied, but homozygous variants predominated in each RNA-Seq experiment (Fig. 2c) as expected from inbred backgrounds [45].

Since the 129P2 inbred strain (used for *Sall2* gene targeting) was already characterized in the Mouse Genome Project (Wellcome Sanger Institute, UK) [46, 47], we next applied the pipeline to identify 129-derived variants from the *Sall2* KO sequencing experiment. We plotted variants from each genotype according to genomic coordinates using our script written in R (genotype_variants.R, Fig. 2d). Variants were binned every 10 million base pairs (Mb) from each genotype and plotted by chromosome. In the case of *Sall2* KO, the distribution of KO common variants was similar to the distribution of WT variants, with the exception of Chr 14, where the *Sall2* gene targeting was done (located at 52.3 Mb) (Fig. 2d). We also investigated the distribution of all variants (subtracting C57BL/6J variants) in each KO line analyzed and applied the Cochran-Armitage test for trend distribution to find chromosomes presenting differential distribution of variants. According to the analysis, the *Gtf2ird1* KO line displayed extensive backcrossing with C57BL/6J and shows a congenic footprint on Chr 5 where the *Gtf2ird1* gene is located (*P* < 0.0001, Cochran-Armitage test for trend distribution) (Additional file 2). The *Mecp2* KO also presented extensive backcrossing with C57BL/6J mice, but not an obvious footprint on Chr X where the *Mecp2* gene is located (*P* = 0.4508) (Additional file 2). Still, variants linked to the targeted gene were expected due to the congenic nature of this KO line.

Similar to the *Gtf2ird1* KO, the *Stc1* KO line presented extensive backcrossing with C57BL/6J and a clear footprint on Chr 14 where *Stc1* is located (*P* < 0.0001) (Additional file 2). The *Itch* KO also presented extensive backcrossing with C57BL/6J mice; however, four chromosomes display obvious targeted locus-linked variants (Chr 2, Chr 9, Chr 10 and Chr 16 with *P* < 0.0001 for the first three and *P* < 0.02 for the last) (see Additional file 2).

The *Sall2* KO presented very similar distribution as shown in Fig. 2d, suggesting that most of the variants in this line come from 129P2-derived ESCs (Additional file 2). Thus, the mixed background with the ESCs was obvious in this KO due to the amount of 129P2 introgressed variants along ten chromosomes, including Chr 14 where *Sall2* and the footprint are located. Five chromosomes presented differential distribution of variants, with Chr 14 showing the lowest *p*-value (Additional file 4: Table S1 ). Similar to the *Sall2* KO, the *Hnrnpd* KO displayed a mixed background, but the average distribution of the variants greatly differed between genotypes (Additional file 2). Although a footprint was present on Chr 5 where *Hnrnpd* is located, the variant distribution was significantly different in 12 other chromosomes (Additional file 4: Table S1 ), likely due to a low number of backcrosses with C57BL/6J. Thus, we expected potentially disturbing passenger mutations from 129S6-derived ESCs (W4) in the *Hnrnpd* KO line [48]. We also reviewed *Casp4* variants on Chr 9, a gene naturally inactivated (5 base pair deletion) in several 129 strains (S1, S2, S6, P2, X1) [49]. Variant calling from every biological replicate of this study revealed the genotype of 129 congenic *Casp4* across samples, evidencing ploidy of *Casp4* 129-derived variants in one WT and in two *Hnrnpd*-KO samples (Additional file 4: Table S2). We confirmed this observation by the lack of expression of *Casp4* exon 7, as described for several 129 strains [50] (Fig. 2e). Thus, besides variants that are linked to the targeted locus, mixed backgrounds in KO lines could have a deep influence on gene expression or phenotypes, as reviewed previously [10, 51, 52].

In addition to the RNA-seq data, we also tested our pipeline using WES data from the GEO dataset, GSE115017, and single cell WGS from the ArrayExpress archive, E-MTAB-4183. We successfully detected the introgressed variants from DBA/2 mice in the C57BL/6J-DBA/2 sample from the GSE115017 study, and mixed background samples from the E-MTAB-4183 study, depicting the number of chromosomes with ESC introgression, respectively (Additional file 3). Taken together, our procedures can offer a reliable way to detect genetic variation from NGS data, effectively identifying genetic introgression.

### Dissection of variants linked to targeted genes: The congenic footprint

**Fig. 3:**
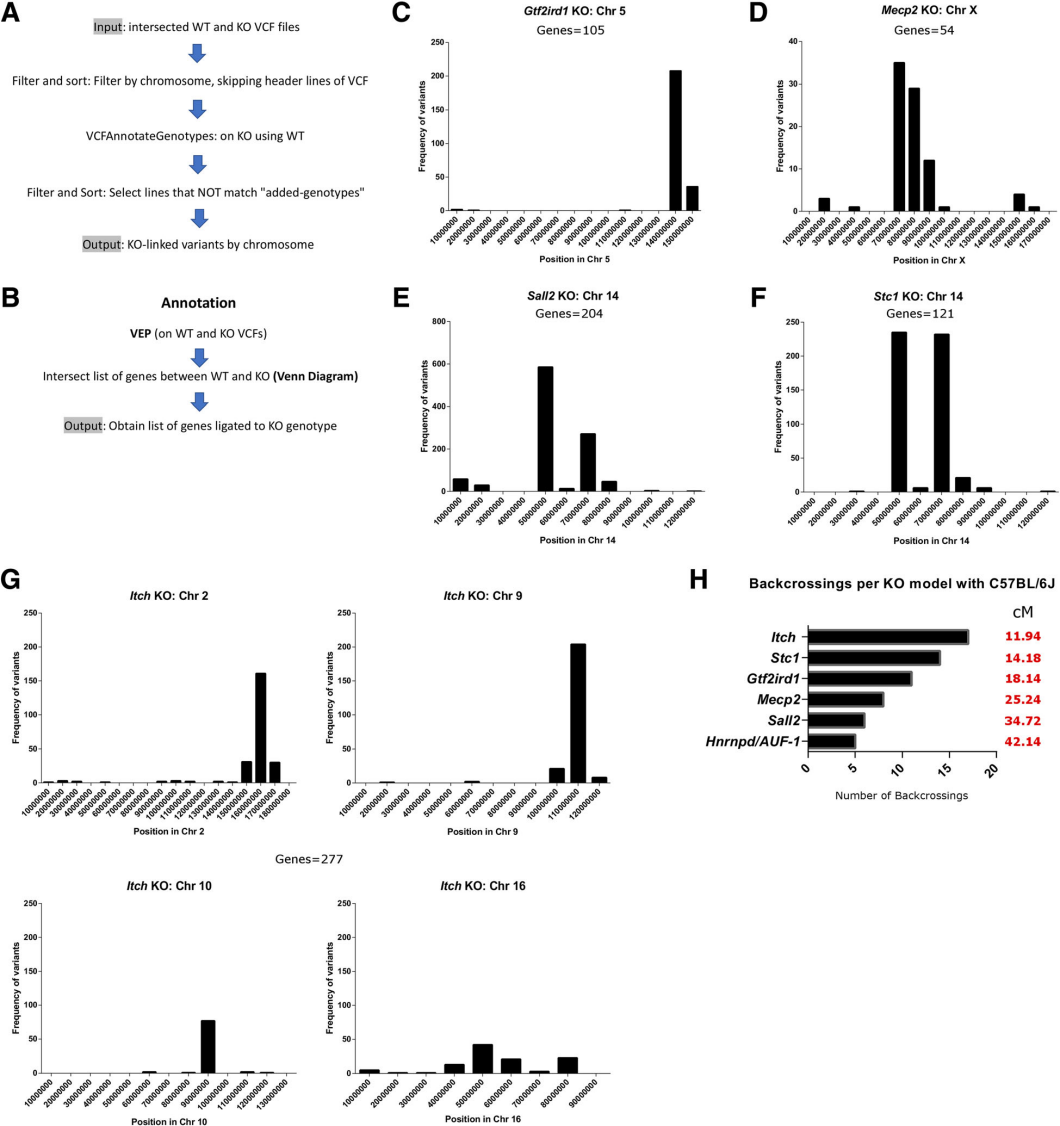
Dissection of variants linked to targeted genes: the congenic footprint. **a** A pipeline for the detection of KO-linked variants based on intersected VCF files per genotype as input. We ignored header lines from the inputted VCF file (Filter and Sort program, skipping header lines starting with #). We performed genotype annotation with the VCFAnnotateGenotypes program on null VCF files using the WT counterpart. After selecting lines that not match “Added-genotypes”, we obtained an output with KO-linked variants. **b** To obtain KO-linked genes from variants in (A), we assessed annotation of variants per genotype with the variant effect predictor program from Ensembl (VEP) and then subtracted KO-linked genes with the aid of Venn diagrams. **c** Histogram of KO-linked variants for the *Gtf2ird1* KO. We binned the genomic coordinates of each chromosome every 10 million bases, and then plotted the variants as frequency histograms according to these positions. The number of congenic genes encompassing these variants is indicated. **d** The same histogram as in (C) for *Mecp2* KO located in Chr X. **e** The same histogram as in (C) for *Sall2* KO located on Chr 14. **f** The same histogram as in (C) for *Stc1* KO located on Chr 14. **g** The same histogram as in (C) for *Itch* KO located on Chr 2. This KO presents four chromosomes with KO-linked variants (Chr 2, 9, 10 and 16). The total number of congenic genes encompassing these variants is indicated. **h** Predicted number of backcrossings with C57BL/6J mice per model. Genomic range of KO-linked variants per model were calculated with the pipeline and translated to centimorgans (cM) with the mouse map converter service (http://cgd.jax.org/mousemapconverter/) according to previously published studies [53, 54]. The extension of KO-linked variants per model in cM is depicted in red

Since the existence of variants linked to targeted loci leads to inaccurate comparisons between WT and KO mice, it is important to detect this bias. Our pipeline in the Galaxy platform (also automatized in the BASH pipeline) allows the analysis of variant distribution and extension, the so-called congenic footprint (Fig. 3a). For the analysis of introgressed variants, we input the intersected VCF files from WT and KO genotypes of *Gtf2ird1*, *Mecp2*, *Stc1* and *Itch* congenic lines, in addition to the *Sall2* KO (MEFs) and Hnrnpd/AUF-1 KO due to the presence of variants on Chr 14 and Chr 5, respectively. We initially performed genotype annotation on KO VCF files using WT VCF files and then selected variants in chromosomes with a significant difference in distribution detected using a Cochran-Armitage test. After selecting lines of KO-annotated VCF files that do not match genotypes, an output with KO-linked variants per chromosome was obtained. For the functional characterization, the variant predictor algorithm (VEP) was used to determine the effect of these variants, including the list of genes linked to the KO genotype (Fig. 3b). As expected, we detected a single chromosome with footprint variants in *Gtf2ird1*, *Mecp2*, *Sall2* and *Stc1* KO lines (Fig. 3c, d, e, and f, respectively) each one with a different number of variants (Additional file 5: Table S1-S4 ). The distributions of variants in the *Sall2* and *Stc1* KOs were similar because both genes are located on Chr 14 (Fig. 3e and f, respectively). Surprisingly, *Itch* KO displayed a footprint in four chromosomes, including Chr 2 where the gene targeting was done (Fig. 3g and Additional file 5: Table S5). We also noticed that variants outside of Chr 2 were heterozygous and could be inherited with the congenic footprinting. VEP annotation led to a diverse number of genes in each KO line (Additional file 5: Table 6). Additionally, the number of backcrossings in each model can be estimated using the formula cM = [200/*N*], where *N* is the generation number and cM the extension of the footprint in centimorgans [53, 54]. The extension of the footprint was estimated with the aid of the histograms in each KO line (denoted as red coordinates in Additional File 5), and homozygous coordinates were transformed to cM using the Mouse Map Converter application (http://cgd.jax.org/mousemapconverter/). Figure 3h depicts the number of backcrossings per KO line, indicating that *Hnrnpd/AUF-1* KO had the lowest number of backcrosses (5) and the largest footprint (42.14 cM), consistent with a mixed background. Conversely, *Itch* KO had the highest number of backcrosses (17) and the smallest footprint (11.94 cM) consistent with a full congenic KO line. These results demonstrate that our novel approach is a reliable method for the detection of introgressed variants and congenic genes, including the estimation of the number of backcrosses in each KO line.

### Ploidy of congenic footprint

**Fig. 4:**
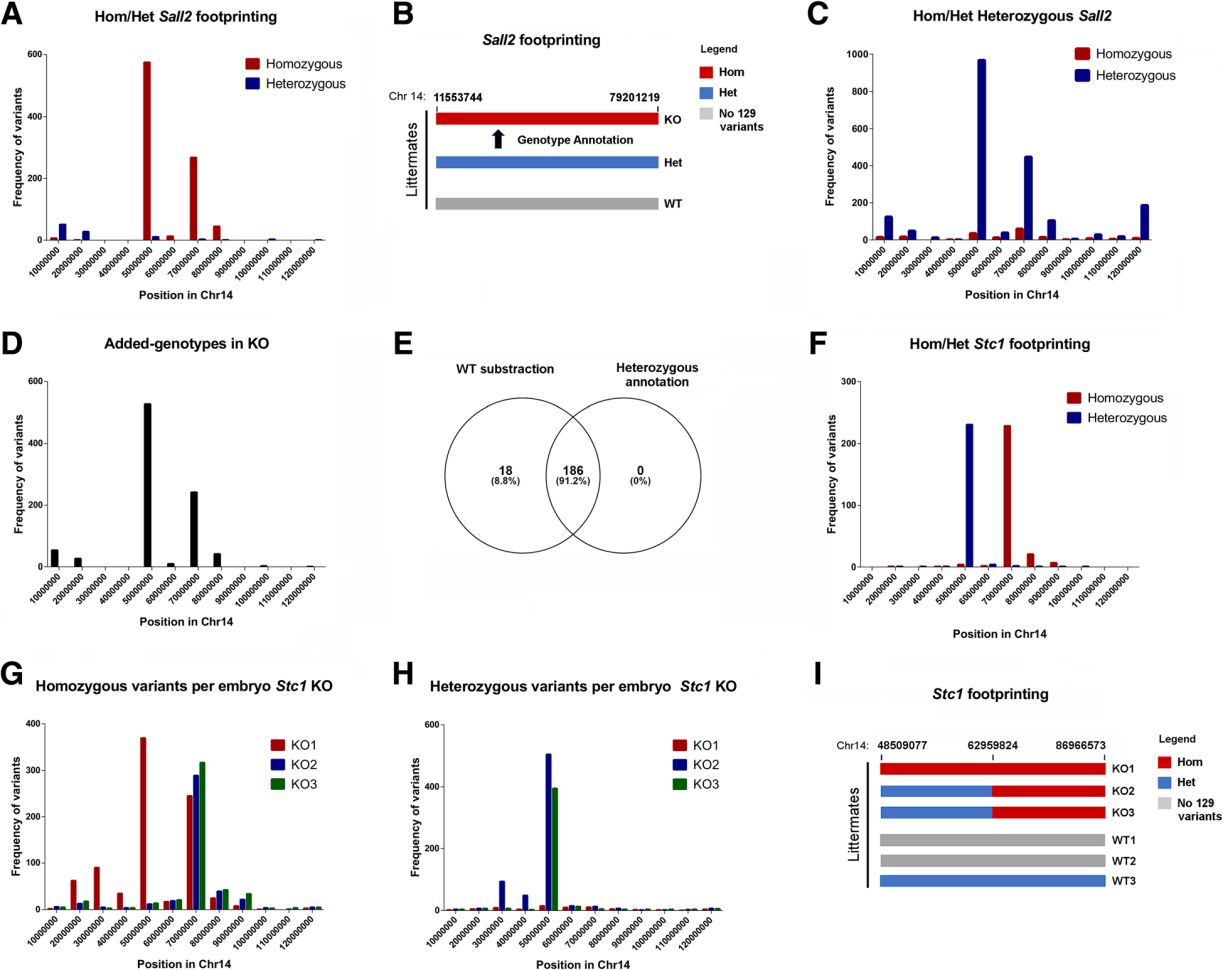
Ploidy of congenic footprint. **a** Histogram of KO-linked variants for *Sall2* KO distinguishing homozygous (red bars) from heterozygous (blue bars) variants. We binned the genomic coordinates of each chromosome every 10 million bases and plotted the variants with each ploidy as frequency histograms according to these positions. **b** Cartoon depicting the congenic footprinting of each embryo in the *Sall2* KO, located on Chr 14. We represented each ploidy with a distinct color (homozygous segment in red, heterozygous segment in blue), marking chromosomal positions above the bars. We performed genotype annotation of KO-linked variants using heterozygous variants from a *Sall2* heterozygous littermate. **c** Histogram of homozygous (red bars) and heterozygous (blue bars) variants from a *Sall2* heterozygous embryo on Chr 14. Variants occurring in the WT embryo were previously subtracted from the heterozygous variants. We plotted the variants with each ploidy as in the graph in (A). **d** Histogram of annotated variants from (A) with heterozygous variants from (B). We plotted the annotated variants as in the graph in (A). **e** Venn diagram comparing the early prediction of congenic genes for the *Sall2* KO (Table S6 in Additional file 5) with annotated variants from (D) showing the overlap. The diagram shows numbers of variants with their corresponding percentages. **f** Histogram of KO-linked variants for the *Stc1* KO differentiating homozygous (red bars) from heterozygous (blue bars) variants. We plotted the variants with each ploidy as in the graph in (A). **g** Histogram of KO-linked homozygous variants per littermate in the *Stc1* KO. We plotted variants from each embryo with a distinct color (KO1 = red, KO2 = blue, KO3 = green). We plotted the variants of each embryo as in the graph in (A). **h** Histogram of KO-linked heterozygous variants per littermate in the *Stc1* KO. We plotted the variants of each embryo as in the graph in (A). **i** Cartoon depicting the congenic footprinting of each littermate in the *Stc1* KO located on Chr 14. We represented each ploidy with a distinct color (homozygous segment in red, heterozygous segment in blue). Chromosomal positions are indicated

We reasoned that the inheritance of congenic variants could vary in ploidy among biological replicates, excluding homozygous inheritance in every case. For this reason, we next addressed ploidy of the congenic footprint in the same KO lines. We focused on the *Sall2* and *Stc1* KOs, as both genes are located on Chr 14. In the *Sall2* KO, 1006 variants were predicted with 80% homozygosity linked to the KO genotype (Additional file 5: Table S3 ). We noticed a center-oriented distribution of these variants (Fig. 4a). To study inheritance of these variants and further characterize the ploidy of the congenic footprint, we used variants from a heterozygous *Sall2* MEF littermate. If the inheritance of the footprint is Mendelian, we reasoned that heterozygous calls in the heterozygous *Sall2* MEF (*Sall2*^*+/−*^) will contain the introgressed variants (Fig. 4b). We found 178 homozygous and 1977 heterozygous variants in the *Sall2*^*+/−*^ MEF littermate (Additional file 6: Table S1 and S2 ) also with center-oriented distribution of the heterozygous variants (Fig. 4c). We used heterozygous variants from these MEFs to annotate genotypes on the previous 1006 predicted *Sall2*-KO linked variants. In agreement with the prediction, 906 variants were annotated using this procedure, covering 91.2% of the *Sall2*-KO introgressed genes (Figs. 4d, e and Additional file 6: Tables S3-S4). Thus, inheritance of the *Sall2*-KO linked variants is homozygous in its extension.

In the case of the *Stc1* KO, nearly half of the variants were heterozygous; thus, the ploidy of this footprint has heterozygous and homozygous distribution (Fig. 4f). Reviewing the distribution of homozygous and heterozygous variants for every littermate showed that the KO1 embryo displayed homozygous variants in both homozygous and heterozygous portions of the footprint, while KO2 and KO3 embryos only displayed these variants at the homozygous portion (Fig. 4g). Conversely, KO2 and KO3 embryos displayed heterozygous variants, while KO1 barely has these types of variants (Fig. 4h). Thus, the KO1 embryo is homozygous for both portions of the footprint while KO2 and KO3 are not. Figure 4i shows a summary of the ploidy in every littermate for the *Stc1* KO line, evidencing ploidy variability in the footprint region. All these analyses suggest that the inheritance of the congenic footprint is complex and cannot be assumed as homozygous in every case.

### The congenic footprint influences gene expression of *Sall2*-KO MEFs

**Fig. 5:**
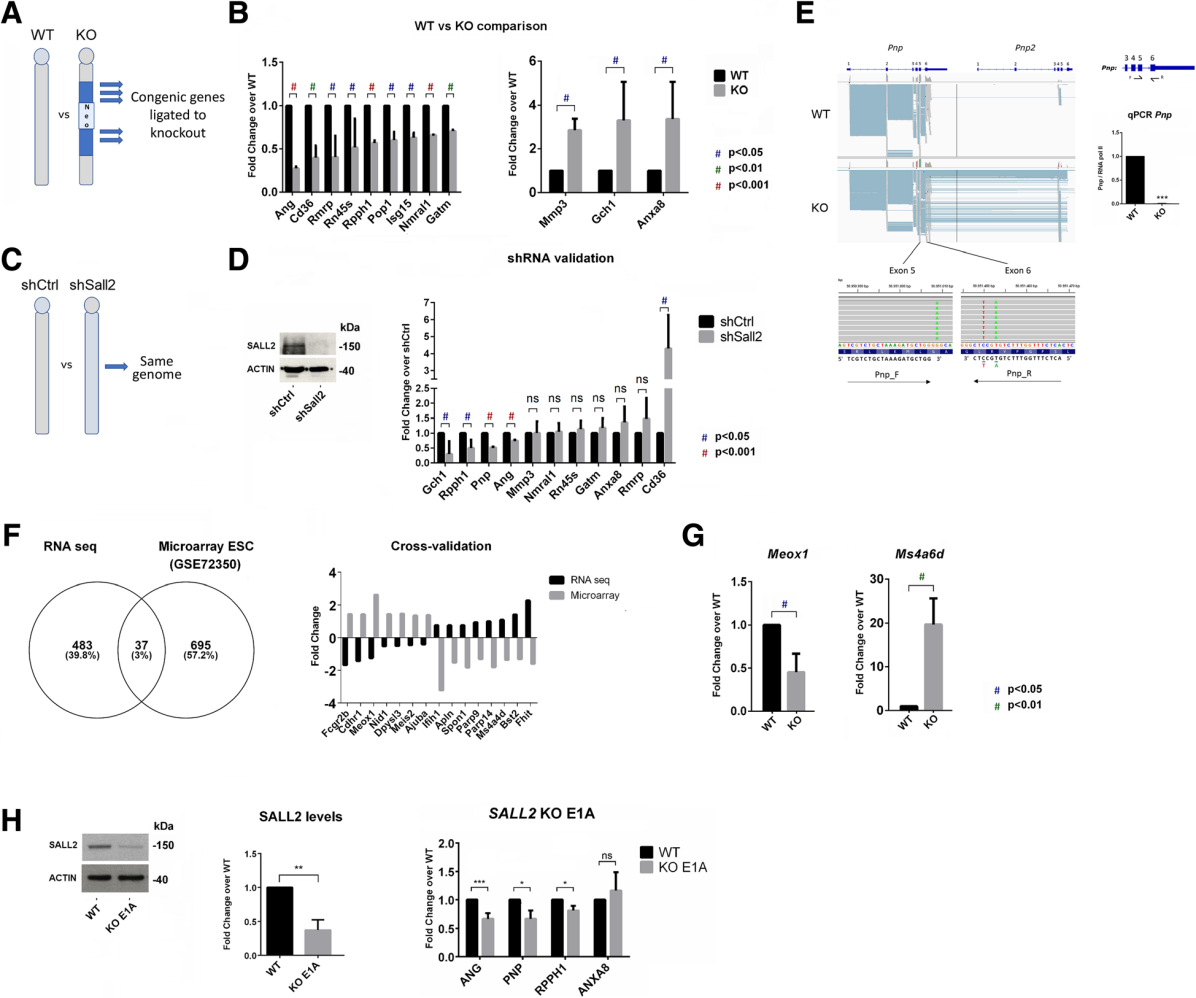
The congenic footprint influences gene expression of *Sall2*-KO MEFs. **a** Cartoon comparing a segment of Chr 14 in *Sall2* WT versus *Sall2*-KO MEFs. Grey color denotes the C57BL/6J recipient strain, and the blue segments represent the ESC introgressed genome with corresponding congenic genes, flanking the *Sall2* locus (Neo = neomycin cassette used in the homologous recombination). **b** Validation of several downregulated (left) and upregulated DEGs (right) between *Sall2* WT and KO iMEFs. We isolated, reverse transcribed and analyzed RNA from *Sall2* WT and KO iMEFs by quantitative real-time PCR. Shown are the expression levels normalized to RNA pol II (*Polr2A* gene) for every gene when compared to levels in WT. We expressed the values as fold change from WT (*N* = 3; data is represented as mean ± s.e.m.), blue # *P* < 0.05; green # *P* < 0.01; red # *P* < 0.001 versus WT; Student’s T-test. **c** Cartoon comparing Chr 14 between scramble (shCtrl) and *Sall2*-silenced cells (shSall2). Grey color denotes the C57BL/6J recipient strain, showing this comparison as genetically correct. **d** *Left:* Representative Western blot for SALL2 and ACTIN in scramble (shCtrl) and *Sall2*-silenced (shSall2) in WT iMEFs. *Right:* Cross-validation of genes in *Sall2*-silenced cells by qPCR as in (B). We normalized the values against RNA pol II (*Polr2A* gene) and expressed them as fold change from scramble (shCtrl) (*N* = 3; data is represented as mean ± s.e.m.), blue # *P* < 0.05; red # *P* < 0.001 versus shCtrl; Student’s T-test. **e** *Left:* IGV snapshot of the WT and congenic *Pnp/Pnp2* gene. The numbers depict exons of both genes. Primers used for detection are indicated in every exon (Exon 5 = Forward, Exon 6 = Reverse). We magnified mismatches from IGV in both primers (in red and green), and we underlined it around the reverse primer in exon 6. *Right:* Schematics of the *Pnp* gene and the position of primers for quantitative real-time PCR. Lower: Quantitative real-time PCR of *Pnp* mRNA in *Sall2* WT and null iMEFs. Shown are the *Pnp* expression levels normalized to *Polr2A* gene for *Pnp* when compared to levels in WT (*N* = 4, *** *P* < 0.001, versus WT; Student’s T-test). **f** *Left:* Venn diagram comparing DEGs from *Sall2* WT versus KO MEFs (FDR < 0.01, Fold Change > 0.35) with DEGs from a microarray of *Sall2* induction in ESCs (Fold Change > 1.3, GSE72350). The overlap reveals 37 common DEGs. *Right:* DEG cross-validation by fold change from the overlap of both studies. We considered only DEGs with opposing fold changes in both studies. **g** qPCR validation of two DEGs (*Meox1* and *Ms4a6d*) from the latter in *Sall2* WT versus null iMEFs (*N* = 3; data is represented as mean ± s.e.m.). blue # *P* < 0.05; green # *P* < 0.01 versus WT; Student’s T-test. **h** Cross-validation of *Sall2*-responsive DEGs in a CRISPR-Cas9 (KO E1A) model in HEK293. *Left*: Representative Western blot for SALL2 and ACTIN in HEK293 individual clones transfected with Control CRISPR (without sgRNA) or SALL2 E1A CRISPR with the quantification of SALL2 protein bands normalized with ACTIN in the bar graph (*N* = 4 for control (WT), N = 4 for CRISPR SALL2E1A (KO E1A), ** *P* < 0.01 versus WT; Student’s T-test.) *Right*: Validation of several downregulated DEGs in the KO E1A model by qPCR. We expressed the values as fold change from WT (*N* = 3; data is represented as means±s.e.m.). *** *P* < 0.001; * *P* < 0.05; ns, non-significant versus WT; Student’s T-test

We then investigated the influence of the congenic footprint on gene expression using *Sall2*-KO MEFs (Fig. 5a). To study the 129P2 genome introgression on Chr 14, we compared the transcriptome of WT versus KO MEFs, identifying 520 DEGs with FDR < 0.01 (Additional file 7: Table S1). Gene ontology analysis showed several clusters of genes involved in immune and inflammatory responses, virus response and cell adhesion, among others, suggesting an association of *Sall2* with these biological functions (Additional file 7: Table S2). We confirmed several DEGs by qPCR, including non-coding genes, such as *Rmrp*, the pre-ribosomal transcript, *Rn45s,* and genes located in the introgressed region, such as *Ang*, *Rpph1*, *Gch1*, and *Anxa8* (Fig. 5b). To address whether these genes are *Sall2*-dependent, we silenced *Sall2* in WT MEFs using a short hairpin RNA (shRNA) and then performed qPCR to examine the impact of *Sall2* silencing within the same genetic background (Fig. 5c). From all the genes tested, only *Gch1*, *Rpph1*, *Ang*, and *Cd36* were affected by *Sall2* silencing, but with different fold changes compared with that obtained using RNA-Seq analysis (see *Pnp* in Fig. 5d). Although qPCR analysis initially showed that *Pnp* was downregulated in *Sall2-*KO MEFs, this result was an artifact resulting from poor reverse primer hybridization due to mismatches in the genomic region, confounding the analysis of *Pnp* in *Sall2*-KO cells (Fig. 5e).

To confirm *Sall2*-dependent DEGs in another genetic background, we also used data from a microarray study of transcription factor (TF)-inducible mouse ESCs in which a single TF (such as *Sall2*) is induced in a doxycycline-controllable manner [55], which allowed cross-validation of 37 other DEGs from the RNA-Seq experiment (Additional file 7: Table S3). From this comparison, 15 DEGs presented similar fold changes between studies (Fig. 5f). We evaluated two of these DEGs by qPCR, confirming trends from the RNA-Seq and the microarray studies (Fig. 5g). These 15 DEGs partly confirmed the initial gene ontology terms (Additional file 7: Table S4). Additionally, we cross-validated the *Sall2*-dependent downregulation of *Ang*, *Pnp*, and *Rpph1* using a CRISPR model of SALL2 in HEK293 cells, lacking the highest expressed isoform of *Sall2* (Fig. 5h). Our study confirmed that the congenic footprint and its interaction with the genetic background influence transcriptome analysis from KO lines. Thus, additional experimental approaches and cross validation are required to determine gene-dependent targets.

### Screening of expression quantitative trait loci (eQTL) in the *Sall2* KO congenic region

**Fig. 6:**
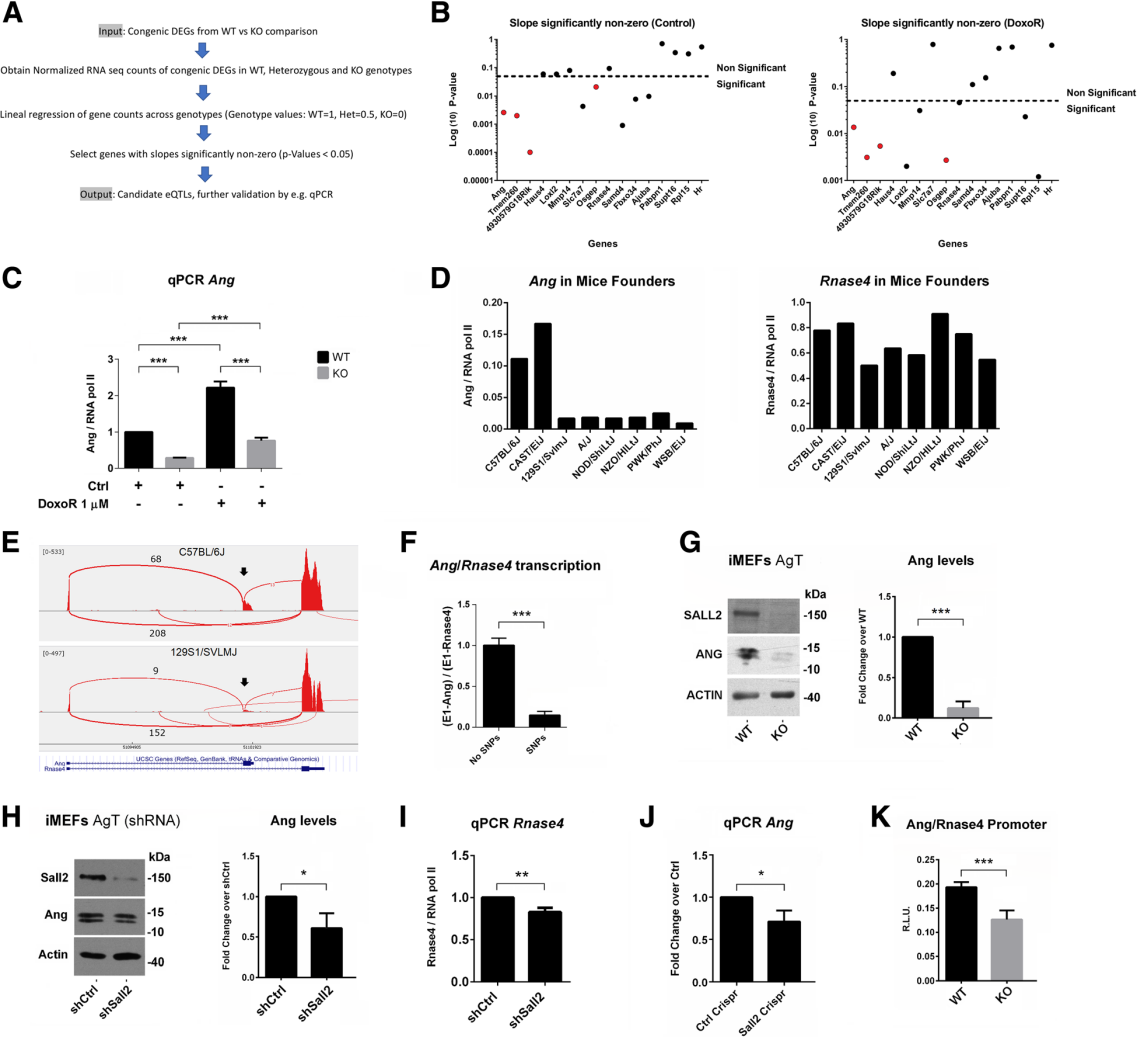
Screening of eQTLs in the *Sall2* KO congenic region. **a** Pipeline based on the linear dependency of gene expression to genotype for the detection of expression quantitative trait loci (eQTL) in congenic regions. Digital counts of gene expression from congenic DEGs in WT, heterozygous and KO genotypes are normalized (e.g. RUVSeq package, available in Bioconductor) and used for further analysis. Then, lineal regression of gene counts across genotypes is obtained, previously defining arbitrary values for each genotype (WT = 1, Het = 0.5, KO = 0). Genes with slopes significantly different than zero are selected according to *P* < 0.05. Finally, an output list of genes with candidate eQTLs is obtained. **b** *Left:* DEGs with significant eQTLs in control after linear regression analysis as described in (A). Genes with slope significantly different from zero are candidate eQTLs and potential modifier genes in the *Sall2* KO. We settled the *P* = 0.05 threshold using a dashed line. *Right:* Same analysis for the congenic DEGs with doxorubicin perturbation. We settled the *P* = 0.05 threshold using a dashed line. We selected candidate DEGs with eQTLs based on significant *P*-values both in control and with doxorubicin perturbation. Four genes were selected for further analysis (*Ang*, *Tmem260*, *4930579G18Rik*, and *Osgep,* denoted as red dots in both graphs). **c** qPCR validation of *Ang* expression in *Sall2* WT and KO MEFs with or without doxorubicin treatment (16 h with 1 μM doxorubicin). Shown are *Ang* expression levels normalized to *Polr2A* gene when compared to levels in WT or doxorubicin treatment. (N = 4; data is represented as means ± s.e.m.). *** *P* < 0.001 versus WT or doxorubicin treatment; Student’s T-test. **d** *Left*: Normalized reads per kilobase per million (RPKM) of Angiogenin (*Ang*) against RNA Pol II (*Polr2A*) across the eight mice founder strains. *Right*: Same analysis as described for the *Rnase4* gene. **e** Sashimi plots of the *Ang/Rnase4* gene expression in C57BL/6J (upper) and 129S1/SvlmJ (lower) strains. Per-base expression is plotted on the y-axis of Sashimi plot; genomic coordinates on the x-axis and the gene structure are represented on the bottom (in blue, obtained from the USCS server). Lower black arrows indicate *Ang* expression and upper black numbers indicate *Ang* junctions. Lower black numbers indicate *Rnase4* junctions. **f** Ratio of *Ang* versus *Rnase4* junctions in strains with (129S1/SvlmJ, A/J, NOD/ShiLtJ, NZO/HILtJ, PWK/PhJ and WSB/EiJ, respectively) and without (C57BL/6J and CAST/EiJ strains) SNPs in the *Ang/Rnase4* locus. **g** *Left:* Representative Western blot for SALL2, ANG, and ACTIN in *Sall2* WT and KO iMEFs. *Right:* Quantification of ANG protein bands normalized with ACTIN in the bar graph (N = 3, *** *P* < 0.001 versus WT; Student’s T-test.). **h** *Left:* The same analysis of (G) for ANG in scramble (shCtrl) and *Sall2*-silenced cells (shSall2). *Right:* Quantification of ANG protein bands normalized with ACTIN in the bar graph at the right (N = 3, *** *P* < 0.001 versus shCtrl; Student’s T-test.). **i** Validation of *Rnase4* levels in the shRNA-silencing model of Sall2 in iMEFs. Shown are the expression levels normalized to RNA pol II (*Polr2A* gene) for *Rnase4* when compared to levels in shCtrl. We expressed the values as fold change from shCtrl (N = 3, * *P* < 0.05 versus shCtrl; Student’s T-test). **j** Validation of *Ang* levels in a CRISPR-Cas9 silencing model of *Sall2* in iMEFs. We expressed the values as fold change from control CRISPR (N = 3, * *P* < 0.05 versus Control CRISPR; Student’s T-test). **k** Luciferase assay with the murine *Ang/Rnase4* promoter electroporated in *Sall2* WT and null iMEFs. We measured luciferase and β-galactosidase activities. R.L.U = relative luminescence units to β-gal (N = 3, *** *P* < 0.001 versus WT; Student’s T-test)

The above methodology did not account for potential modifier genes acting as eQTL from the congenic region of KO mice. In the case of the *Sall2*-KO MEFs, about 204 genes on Chr 14 were congenic; therefore, we expected genetic interference with the KO mutation (Additional file 5: Table S6). Out of the 204 genes, 61 carried missense mutations, and 17 were DEGs (Additional file 8: Table 1). To identify potential modifier genes in the congenic region of the *Sall2* KO, we applied the eQTL strategy [56]. We propose a pipeline for the detection of candidate eQTLs, based on the linear dependence between expression and genotype, as previously described [57]. We obtained normalized gene counts of congenic genes from *Sall2* WT as well as heterozygous and KO MEFs and tested linear regression against arbitrary values for each genotype (WT = 1, Het = 0.5, and KO = 0) (Fig. 6a).

We also analyzed gene expression using doxorubicin as an environmental perturbation, since this drug increases nucleosome turnover around the promoters of active genes [58]. We tested 16 congenic DEGs ranked by fold change for genotype dependency in the control condition, of which eight display linear genetic dependency (Fig. 6b**, left**). Global perturbation with doxorubicin altered fold changes of these genes and the DEGs with genetic dependence (Fig. 6b**, right**). Four of these genes displayed genetic dependence in both control and doxorubicin-treated conditions (*Ang*, *Tmem260*, *4930579G18Rik* and *Osgep*, see red dots in Fig. 6b), and *Ang* was one of the most differentially expressed genes in both cases (Additional file 7: Table S1 and Additional file 8: Table S2, respectively). *Sall2*-KO *Ang* displayed low expression levels both in control and doxorubicin-treated MEFs compared to WT *Ang* expression. However, the fold change in *Ang* expression induced by doxorubicin was similar between genotypes (Fig. 6c). These results suggest that the congenic (129P2) *Ang* promoter, controlling both *Ang* and *Rnase4* genes [59] is functional, but *Ang* transcription is low in the 129P2 strain. In agreement with our data, RNA-Seq data from the striatum of the eight Collaborative Cross founder strains [60] (SRA project ID: PRJNA228935) showed that *Ang* expression is remarkably low in six out of the eight strains (except C57BL/6J and CAST/EiJ), values corresponding to outliers in comparison to the group. We did not see this effect in the expression of *Rnase4* (Fig. 6d). Moreover, strains with low levels of *Ang* in the striatum presented several variants in the *Ang/Rnase4* gene, which were absent in the C57BL/6J and CAST/EiJ strains (Additional file 9A). These variants are also present in *Sall2*-KO MEFs, congenic from 129P2, but absent in the WT counterpart (Additional file 9B), suggesting an association of these variants with the low expression of congenic *Ang*. In line with this, Sashimi plots from the RNA-Seq data across mice founders supported by-pass of *Ang* transcription linked to the genomic variants (Fig. 6e and f, respectively and see Additional file 9C). Furthermore, an independent RNA-Seq study from the hippocampus of 129S1/SvImJ mice [61] (GEO DataSets accession GSE76567) showed strong downregulation of *Ang* transcripts compared to the C57BL/6J mice (Additional file 9D), a trend that we also experimentally confirmed in the cortex of the *Sall2*-KO mice by qPCR (Additional file 9E). By Western blot analysis, we confirmed strong downregulation of ANG protein levels in *Sall2*-KO MEFs (Fig. 6g), in agreement with the low *Ang* early detected by qPCR (See Ang in Fig. 5b). In contrast, mild downregulation of ANG protein levels was detected in *Sall2-*silenced cells (Fig. 6h) along with mild downregulation of *Rnase4* (Fig. 6i). Similarly, CRISPR-Cas9-mediated *Sall2*KO in WT MEFs showed mild downregulation of *Ang* (Fig. 6j, see model validation in Additional file 11). These results suggest that SALL2 transcriptionally regulates *Ang*/*Rnase4*, but *Ang* expression is additionally affected by congenic variants present in the *Sall2* KO line. Consistent with transcriptional regulation by *Sall2*, the *Ang/Rnase4* promoter contains a cluster of three SALL2 binding sites around the transcription start site (data not shown). An *Ang/Rnase4* promoter of 1231 base pairs displayed less activation in *Sall2*-KO versus WT cells, consistent with the mild downregulation of *Ang* and ANG protein levels in *Sall2*-silenced cells (Fig. 6k). Taken together, congenic *Ang* is transcribed at low levels due to genetic determinants inherited from 129P2, somehow masking *Sall2*-dependent transcriptional regulation. Thus, *Ang* could be classified as a potential modifier gene in *Sall2*-KO MEFs.

### Genetic interference of *Cdkn1a*, a canonical target of *Sall2*

**Fig. 7:**
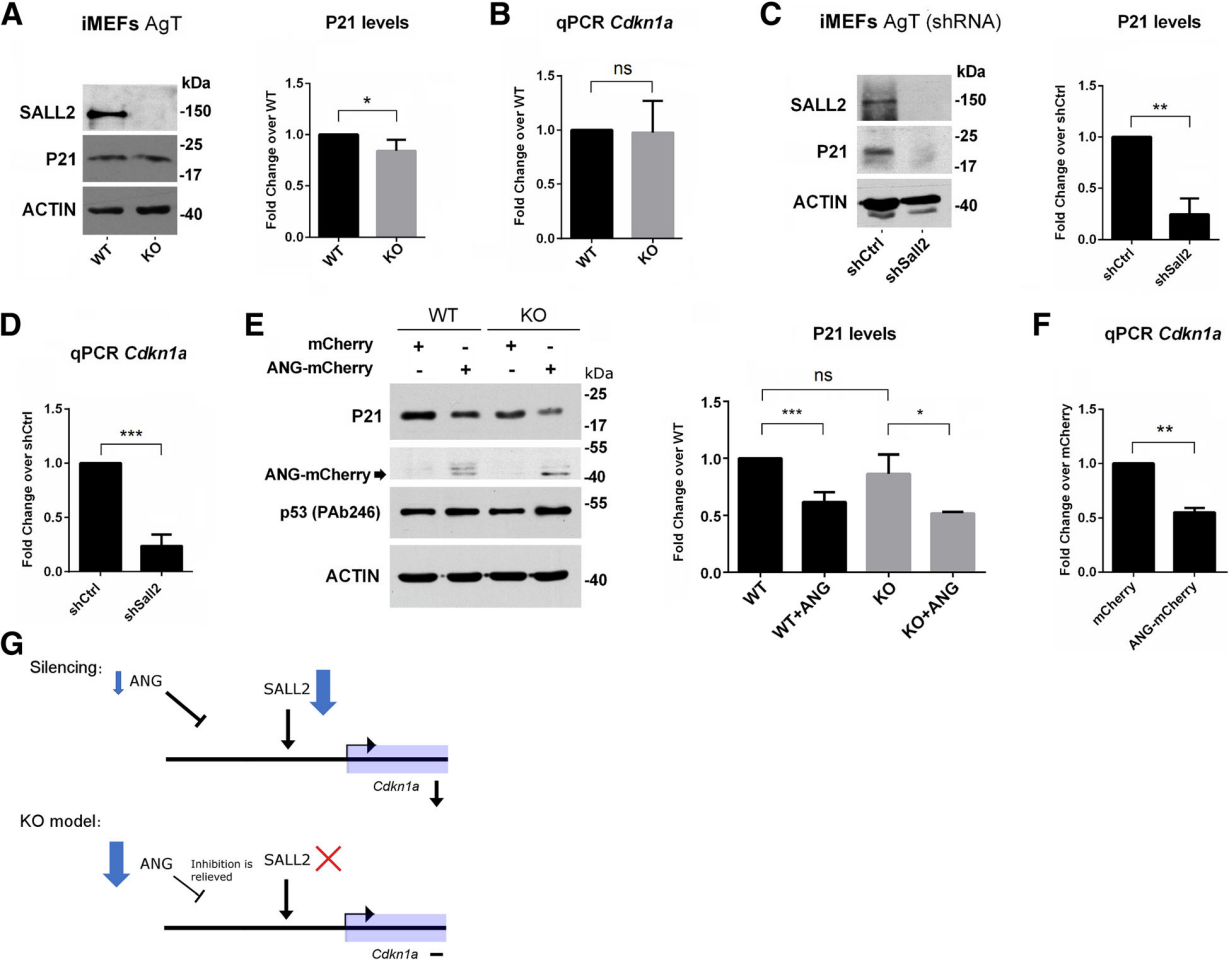
Genetic interference of *Cdkn1a*, a canonical target of Sall2. **a** *Left:* Representative Western blot for SALL2, P21, and ACTIN in *Sall2* WT and KO cells. *Right:* Quantification of P21 protein bands normalized to ACTIN (N = 3, *** *P* < 0.001 versus WT; Student’s T-test.). **b** *Cdkn1a* mRNA levels in *Sall2* WT versus KO iMEFs. We isolated, reverse transcribed and analyzed RNA from WT or *Sall2*-null iMEFs by quantitative real-time PCR. Shown are the expression levels normalized to the *Polr2A* gene for *Cdkn1a* when compared to levels in *Sall2* WT iMEFs. We expressed the values as fold change from WT iMEFs (N = 3, * *P* < 0.05 versus WT; Student’s T-test). **c** *Left:* Same analysis as in (**a**) from scramble (shCtrl) and *Sall2*-silenced cells (shSall2). *Right:* Quantification of P21 protein bands normalized to ACTIN (N = 3, *** *P* < 0.001 versus shCtrl; Student’s T-test). **d**) Same analysis as in (**b**) for *Cdkn1a* mRNA levels in scramble (shCtrl) versus *Sall2*-silenced iMEFs (shSall2). We expressed the values as fold change from shCtrl iMEFs (N = 3, *** *P* < 0.001 versus shCtrl; Student’s T-test). **e**) *Left:* Representative Western blot for P21, ANG-mCherry, P53, and ACTIN in *Sall2* WT and KO iMEFs. Where indicated, we electroporated plasmids encoding mCherry or ANG-mCherry. After 16 h, the lysates were analyzed. *Right:* Quantification of P21 protein bands normalized to ACTIN (N = 4 for WT, N = 3 for KO, *** *P* < 0.001, * *P* < 0.05, ns = non-significant versus WT or KO; Student’s T-test.). **f** RNA from *Sall2* WT iMEFs electroporated with mCherry or ANG-mCherry for 16 h were isolated, reverse transcribed and analyzed by quantitative real-time PCR. Shown are the expression levels of *Cdkn1a* normalized to *Polr2A* (N = 3; data is represented as mean ± s.e.m.). *** *P* < 0.001 versus mCherry; Student’s T-test. **g** Model of *Cdkn1a* regulation based on SALL2 and ANG as opposite regulators. *Upper:* In the *Sall2*-silencing model, minor downregulation of the repressor of *Cdkn1a* (ANG, small blue arrow) and strong downregulation of the activator (SALL2, enlarged blue arrow) downregulates *Cdkn1a* mRNA. *Lower:* In the *Sall2* KO model, the activator of *Cdkn1a* (SALL2, red cross) is absent, consequently, the repressor (ANG, enlarged blue arrow) expresses at very low levels, relieving the repression of *Cdkn1a* mRNA

As an example of how introgressed genes can act as gene expression modifiers, we focused on *Cdkn1a* (p21^CIP/WAF^), a gene known to be regulated by both SALL2 and ANG. SALL2 is known to induce *Cdkn1a* in neurons, ovarian epithelial cells and MEFs under genotoxic stress [33, 62, 63]. On the other hand, ANG negatively regulates p21^CIP/WAF^ through p53 degradation in human cells [64, 65]. *Cdkn1a* was not detected as a DEG in the RNA-Seq analysis by comparing *Sall2* WT vs KO MEFs. Therefore, genetic interference with *Cdkn1a* transcription is likely to occur in *Sall2-* KO mice due to the existence of a congenic footprint. We noticed minor changes in p21 protein and *Cdkn1a* mRNA levels between *Sall2* WT and KO MEFs (Fig. 7a and b, respectively). Conversely, shRNA-mediated *Sall2* silencing in *Sall2* WT MEFs showed strong downregulation of p21 protein and *Cdkn1a* mRNA levels, consistently with previous reports [63, 66] (Fig. 7c and d, respectively). We hypothesized that the downregulation of ANG partly explains the unchanged p21 protein and *Cdkn1a* mRNA levels in *Sall2*-KO MEFs. In agreement with this hypothesis, mature ANG expression into *Sall2* WT MEFs readily downregulated p21 protein and *Cdkn1a* mRNA levels, assuming transcriptional repression of *Cdkn1a* by ANG (Fig. 7e and f, respectively). Surprisingly, we did not see this effect on endogenous P53 protein levels (TRP53), as previously reported [64]. A model of *Cdkn1a* regulation was proposed based on SALL2 and ANG as opposite regulators. In the *Sall2*-silencing model, mild downregulation of ANG protein levels is achieved; thus, the activator of *Cdkn1a* (SALL2) is in very low levels while the repressor (ANG) is present, downregulating *Cdkn1a* (Fig. 7g, upper). Conversely, in the *Sall2* KO model, the activator is lost, and the repressor is expressed at low levels, relieving the inhibition on *Cdkn1a* (Fig. 7g, lower). These suggest that ANG exerts transcriptional regulation on *Cdkn1a* in a manner that opposes SALL2, interfering *Cdkn1a* regulation in *Sall2*-KO cells.

## Discussion

The origin of the ESCs used in gene targeting, the number of backcrosses and consecutive breeding used for the maintenance of GEM (KO/KI) lines (including potential genetic drift) all can have a profound impact in the genetic make-up of these models. These genetic variations within mice from the same KO or KI line will influence gene expression and phenotypes, potentially jeopardizing experimental conclusions. Thus, the genetic background of GEM mice imposes biases that need to be addressed before making conclusions to ensure reproducibility of gene expression and the phenotypes associated to a targeted gene.

We designed an automatized pipeline implemented in both the Galaxy platform and in a BASH/R script to perform genetic background characterization of GEM lines. Using NGS data, our pipeline can 1) identify introgression of ESC-derived variants in the C57BL/6 background and other recipient genomes, including genome-wide variant visualization; 2) define partial congenic, fully congenic, or mixed backgrounds and 3) detect and characterize the ploidy of the congenic footprint. After applying the pipeline, the Ensembl variant predictor algorithm [44] can be used to classify variants as novel or existent. However, a potential limitation of our pipeline in Galaxy, using WGS data (at high depth) is the amount of computational time employed in the variant calling, making the use of public servers impractical and restricting the calculations to a cluster. To circumvent this problem, we implemented the pipeline purely in BASH, raising the open file limit for such analysis (see Github: https://github.com/cfarkas/Genotype-variants). Thus, our pipeline is flexible in the use of both RNA and DNA sequencing data. Large-scale genomic sequencing data is superior for measuring introgression of genes or genomic segments, from one strain to another, as well as for identifying sequence differences in non-transcribed DNA. However, using RNA seq data, it is possible to assess influences on gene expression caused by the congenic footprints and to identify putative modifier genes with an eQTL strategy. Of relevance, is that our approach provided the opportunity to uncover genetic contamination along with novel variants fixed by genetic drift.

SNP genotyping panels are currently used to perform genetic background characterization; however, there are disadvantages in using this approach for genetic background characterization of GEM lines. A summary of the advantages and disadvantages of SNP genotyping and the pipeline are presented in Table 1**.** For example, our pipeline, which is based on NGS sequencing data, is versatile (accepts WGS/WES/RNA-Seq data) and greatly outperforms dense SNP arrays in sensibility and detection of novel/rare variants (especially DNA-Seq data [67]). Furthermore, it allows visual estimation of the extension of variants with the aid of histograms containing variant frequency per chromosome. As reported, SNP arrays have fixed sensibility and do not perform well in detecting rare variants, including novel variants [68].

**Table 1:** Advantages and Disadvantages for the use of NGS data in genetic background characterization

	Genotype Variants Pipeline	SNP genotyping
Implementation	NGS data: WGS/WES/RNA-seq	Only microarrays
Computational Resources	High for WES/WGS	Low
Rare Variant Detection	Yes	Difficult
Novel variant discovery	Yes	No
QTL detection	Yes, Using RNA-Seq (eQTLs)	Yes, using genetic linkage studies.
Sensibility	Increasing power with more depth	Fixed
Visualisation	Genome wide-plots in R (free)	Programatically or included in commercial programs

We reviewed the advantages and disadvantages in genetic monitoring using NGS data (through our pipeline) versus SNP-dense microarrays**.** We considered implementation, computational resources, rare variant detection, novel variant discovery, QTL detection, sensibility and visualization in each method

To explore the introgression of gene variants in GEM mice, we applied the pipeline using publicly available high throughput data, in addition to our experimental data from *Sall2* KO mice. As a proof of concept, we were able to identify the ploidy of 129-derived variants that leads to a *Casp4* null mutation (reported in several 129 strains) in the background of *Hnrnpd* KO mice. We also found that the number of novel variants is highly variable between KO lines, even overpassing ESC introgressed variants. This observation represents a bias since novel and missense variants correlate in number, imposing novel backgrounds for the KO lines and the need for proper characterization of these variants.

Our studies indicate that the number of congenic genes varies between KO lines, and in one case introgressed genes are outside the targeted chromosome (e.g., for the *Itch* KO). The latter example implies that both genotypes (WT/KO) were independently maintained. Alternatively, we may have detected a partially (incomplete) congenic strain with residual segments outside the targeted chromosome. After obtaining linked variants by the WT subtraction, we suggest DNA sequencing of cells or tissues from heterozygous littermates, as it will further confirm the extension of the footprint. Since most of the variants near the target gene are homozygous, calls from a heterozygous genotype can discriminate these variants assuming Mendelian inheritance. This method was successful in the *Sall2* KO, as evidenced by the > 60 Mb footprint. Nevertheless, a more complex scenario of ploidy can be found, as it is the case of the *Stc1* KO where nearly half of the footprint is heterozygous and introgressed with different ploidy among KO littermates. We recognized that this issue is concerning in terms of reproducibility across biological replicates in KO studies.

Using *Sall2* KO as a model, it was possible to assess the influences on gene expression caused by the congenic footprint and to identify putative modifier genes (eQTLs) using RNA-Seq data. By silencing *Sall2* (using shRNA, CRISPR-Cas9) within cells of same genetic background (WT littermate), we also demonstrated the importance of validation of target-dependent genes initially identified using the *Sall2* WT/KO MEFs. Likely because of the influence of the introgressed 129P2 genome in Chr 14 of *Sall2* KO cells, several DEGs found in WT/KO MEFs comparison could not be confirmed by *Sall2* shRNA experiments. Interestingly, *Pnp*, a gene within the congenic region of Chr 14, was identified as a DEG in the *Sall2-*shRNA studies, but not in the analysis of the RNA-seq data from *Sall2* WT*/*KO lines. Further analysis uncovered a bias caused by genetic variation in the KO model due to mismatches in the PCR primer hybridization region (Fig. 5e). The congenic nature of *Pnp* likely explains the failure to detect it as a DEG in the *Sall2* KO MEFs. In fact, polymorphisms in gene regulatory regions can modify their transcriptional output by creating or ablating transcription factor binding sites or other transcriptional regulatory elements [25].

Using several experimental approaches, we found that the low transcription of *Ang* in *Sall2* KO MEFs is likely caused by genetic components inherited from the targeted ESCs, but also by the absence of functional SALL2 transcription factor. Our experimental data also suggest that congenic *Ang* is a modifier gene, which show effects on genes related to the targeted gene, specifically affecting SALL2-target *Cdkn1a* (p21) expression. However, we cannot discount the idea that the levels of *Cdkn1a* in the *Sall2* KO could be consequence of a polygenic effect and not only due to low levels of ANG.

In summary, due to the mentioned constraints in the use of KO/KI congenic mice, conclusions related to gene expression and phenotypes could be misleading. Selection of an appropriate strain and characterization of the genetic background are critical aspects of any experiment using GEM lines. Even for technical reasons, polymorphisms in coding genes should be detected for adequate primer design if qPCR validation is intended. In silico characterization of variants coming from the genetic background, including the dissection of congenic variants, can improve our understanding of phenotypic outcomes in GEM lines. However, validation of data using alternative approaches (e.g., shRNA, siRNA, and CRISPR-Cas9 targeting) is also required for specific target-dependent conclusions. We suggest generating KOs by genome editing technologies, such as CRISPR-Cas9, in order to assign gene expression and phenotypes solely due to the targeted gene. Nevertheless, genetic characterization is also needed due to the occurrence of off-target mutations or genetic drift. Our strategy can refine the use of KO lines and open opportunities to uncover new genetic interactions, such as the *Ang*/*Cdkn1a* axis described here.

## Conclusions

We present a computational pipeline implemented in the Galaxy platform and in BASH/R script to determine genetic introgression of GEM models using NGS data. The pipeline allows identification of congenic strains, ploidy nature of variants and the estimation of the backcrossing state in the models in use as well as visual assessment of congenic regions in the mouse genome. In addition, it allows identification of putative modifier genes. We suggest that our strategy together with target validation experiments refines the use of KO/KI lines and opens opportunities to uncover new genetic interactions that could impact phenotypic outcomes.

## Additional files

Additional file 1: Schematic view of the sources of genetic variation identified in KO/KI congenic mice. First, the choice of embryonic stem cells (ESCs) derived from the inbred brown mice is a major source of genetic variation, and genetic characterization of these cells could be unavailable (1). Second, the number of backcrossing with C57BL/6 (substrain N or J) will lead to variable introgression of variants from ESCs into littermates, depending on how many backcrossings were performed (2). Finally, further breeding strategies across generations will determine the final constitution of variants in WT and KO/KI littermates, including the congenic footprint (3). The N of crossings will also determine the number of novel fixed variants in animals. Mice images were designed by the authors. (TIF 338 kb)

Additional file 2: Whole genome histogram of novel/existing variants in *Gtf2ird1, Mecp2, Stc1, Itch, Sall2 and Hnrnpd/AUF-1* KO (RNA-Seq). RNA-Seq samples from *Gtf2ird1*, *Mecp2, Stc1, Itch, Sall2 and Hnrnpd/AUF-1* WT and KO embryos were plotted, including WES samples from GSE115017 (GEO datasets) and E-MTAB-4181 (ArrayExpress). We binned the genomic coordinates of each chromosome every 10 million bases, and plotted the variants of each genotype/condition as frequency histograms according to these positions. In the case of RNA-Seq samples, blue bars represent average variants from WT embryos, and red bars represent the average variants from KO embryos in each case. The biological replicates were as follows: In the *Sall2* KO, WT = 1 and KO = 3, in the *Itch* KO, WT = 2 and KO = 2 and in the four other studies, WT = 3 and KO = 3. (PDF 76 kb)

Additional file 3: Whole genome histogram of novel/existing variants in two WES studies. WES samples from the GEO datasets, GSE115017 and from the SRA archive E-MTAB-4181, were plotted as in Additional file 2. The samples selected from the first study were GSM3163042 (C57BL/6J) with GSM3163051 (C57BL/6J mixed with DBA2) and SAMEA3940161 (Tumor1) with SAMEA3940166 (Tumor6) for the second study. A Cochran-Armitage test was included after every plot. (PDF 38 kb)

Additional file 4: **Table S1.** Cochran-Armitage test for trend distribution in knockouts (*p*-values) **Table S2.** *Casp4* variants per biological replicate in the *Hnrnpd* knockout experiment. (XLSX 19 kb)

Additional file 5: **Table S1-S5.** KO-linked variants in *Gtf2ird1*, *Mecp2*, *Stc1* and *Itch* knockout studies, including a *Sall2* KO sequencing experiment. **Table S6.** corresponding congenic genes for the referred KO lines. (XLSX 364 kb)

Additional file 6: **Table S1.** Homozygous variants from a *Sall2* ^+/−^ littermate used to crossvalidate the congenic footprint in *Sall2* KO. **Table S2.** heterozygous variants of the latter embryo. **Table S3.** KO-linked variants annotated with the heterozygous calls from Table S2. **Table S4.** *Sall2* KO congenic genes in the footprint of this line in Chr 14. (XLSX 431 kb)

Additional file 7: **Table S1.** DEGs between *Sall2* WT and *Sall2* KO (FDR < 0.05). *Ang*, one of the most downregulated genes in the *Sall2* KO line is depicted in red. **Table S2.** List of GO terms obtained with InnateDB from DEGs from Table S1. **Table S3.** Overlap between the RNA-Seq study and a Microarray Study of Sall2 induction in ESC. **Table S4.** List of the GO terms obtained with InnateDB from the cross-validated list in Table S3. (XLSX 86 kb)

Additional file 8: **Table S1.** List of congenic DEGs in the *Sall2* KO line (MEFs). Congenic DEGs with missense mutations are depicted in red. **Table S2.** DEGs between *Sall2* WT and KO MEFs under doxorubicin perturbation (FDR < 0.05). *Ang*, one of the most downregulated genes in the *Sall2* KO line, is depicted in red. (XLSX 76 kb)

Additional file 9: Pervasive downregulation of *Ang* in 129 mice. A) *Left*: IGV snapshot of *Ang/Rnase4* gene expression across mouse founders (PRJNA228935 accession). C57BL/6J and 129S1/SvImJ strains are placed in the upper panels. The gene model is shown in blue and was obtained from the UCSC server. B) Same snapshots as in (A) across *Sall2* RNA-Seq samples. C) Sashimi plots of samples in (A) depicting exon usage as the number of junctions. Per-base expression is plotted on the y-axis of Sashimi plot; genomic coordinates on the x-axis, and the gene structure are represented on the bottom (in blue, obtained from the USCS server). D) Gene counts of *Ang* from the hippocampus of C57BL/6J and 129S1/SvImJ mice normalized against *Polr2a* gene counts (GSE76567, *N* = 6, *** *P* < 0.001, versus C57BL/6J; Student’s T-test). E) Quantitative real-time PCR of *Ang* in the cortex coming from *Sall2* WT and null mice. RNA from *Sall2* WT and null cortex were isolated, reverse transcribed and analyzed by quantitative real-time PCR. Shown are *Ang* expression levels normalized to *Polr2A* when compared to levels in WT. (*N* = 3; data is represented as means ± s.e.m.) *** *P* < 0.001 versus WT; Student’s T-test. (TIF 382 kb)

Additional file 10: List of primers used for quantitative real time PCR. (XLSX 10 kb)

Additional file 11: Validation of the murine *Sall2* gene deletion by CRISPR-Cas9. A) Representative Western blot for SALL2 and ACTIN in control and *Sall2*-silenced cells by CRISPR (mSall2 CRISPR) done in *Sall2* WT iMEFs. B) We designed a double CRISPR cut to delete a segment of the *Sall2* gene. The two CRISPRs (denoted as gRNA one and two) targeted the largest exon of the murine *Sall2* gene (exon 2). C). iMEF cells were electroporated with Control CRISPR plasmid or the two mSall2 CRISPR plasmids, and fluorescent cells were enriched by flow-cell cytometry (top 5% of fluorescent cells). We identified the desired deletion from the genomic DNA of a pool of iMEF cells and targeted it with the double CRISPR strategy (amplicon at 500 base pairs in mSall2 lane, denoted with a black arrow). D) Alignment from the Sanger sequencing results of the gel-purified amplicon from (C), depicting the genomic deletion of the *Sall2* gene (chromosomal position 52,314,428–52,315,642 on the mm10 build). We highlighted the codifying sequences of the exon two of murine *Sall2* gene in yellow. (TIF 808 kb)

## Abbreviations

129P2: 129P2OlaHsd mouse strain
Ang: Angiogenin
CRISPR: Clustered regularly interspaced short palindromic repeats
DNA: Deoxyribonucleic Acid
eQTL: Expression QTL
ESC: Embryonic stem cell
GEM: Genetically engineered mice
GEO: Gene Expression Omnibus
iMEFs: Immortalized MEFs
Indel: Insertion/deletion polymorphism
KI: Knock-in
KO: Knockout
MEFs: Mouse embryonic fibroblasts
MGP: Mouse Genomes Project
NGS: Next Generation Sequencing
p21: p21Cip1/Waf1
PCR: Polymerase chain reaction
qPCR: Quantitative real-time PCR
QTL: Quantitative trait loci
RNA-Seq: RNA-sequencing
shRNA: Short hairpin ribonucleic acid
SNP: Single nucleotide polymorphism
SRA: Sequence Read Archive
Trp53: Tumor Protein p53 (mouse)
UCSC: University of California Santa Cruz
VCF: Variant Call Format
VEP: Variant effect predictor
WES: Whole Exome Sequencing
WGS: Whole Genome Sequencing
