# Supplementary figures and images for: Streamlined computational pipeline for genetic background characterization of genetically engineered mice based on next generation sequencing data
Source: BMC Genomics. 2019 Feb 12;20:131. doi: 10.1186/s12864-019-5504-9 (PMC6373082; doi:10.1186/s12864-019-5504-9)

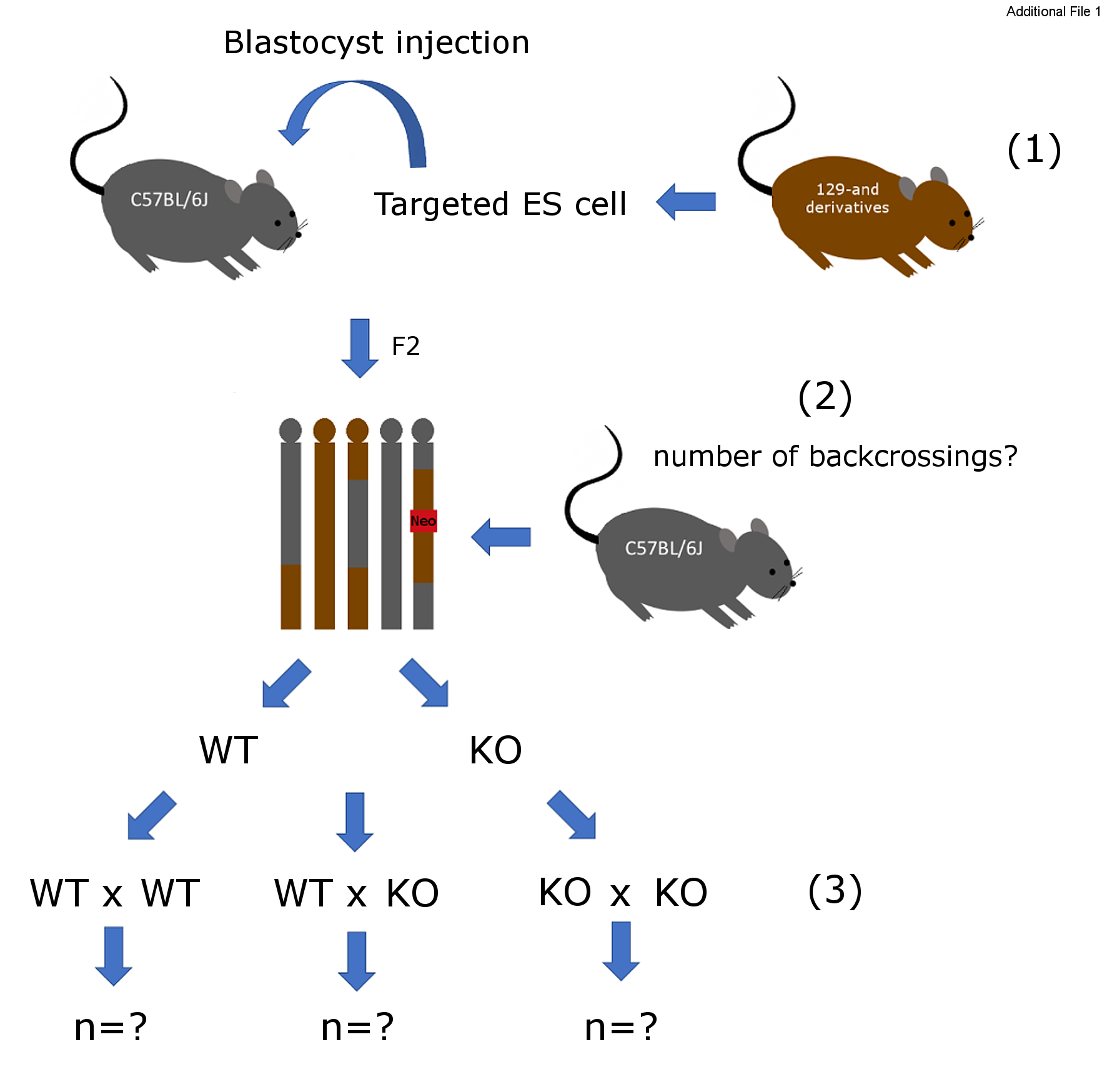

Supplement: Supplementary file 1 — Schematic view of the sources of genetic variation identified in KO/KI congenic mice. First, the choice of embryonic stem cells (ESCs) derived from the inbred brown mice is a major source of genetic variation, and genetic characterization of these cells could be unavailable (1). Second, the number of backcrossing with C57BL/6 (substrain N or J) will lead to variable introgression of variants from ESCs into littermates, depending on how many backcrossings were performed (2). Finally, further breeding strategies across generations will determine the final constitution of variants in WT and KO/KI littermates, including the congenic footprint (3). The N of crossings will also determine the number of novel fixed variants in animals. Mice images were designed by the authors. (TIF 338 kb) [file 12864_2019_5504_MOESM1_ESM.tif]

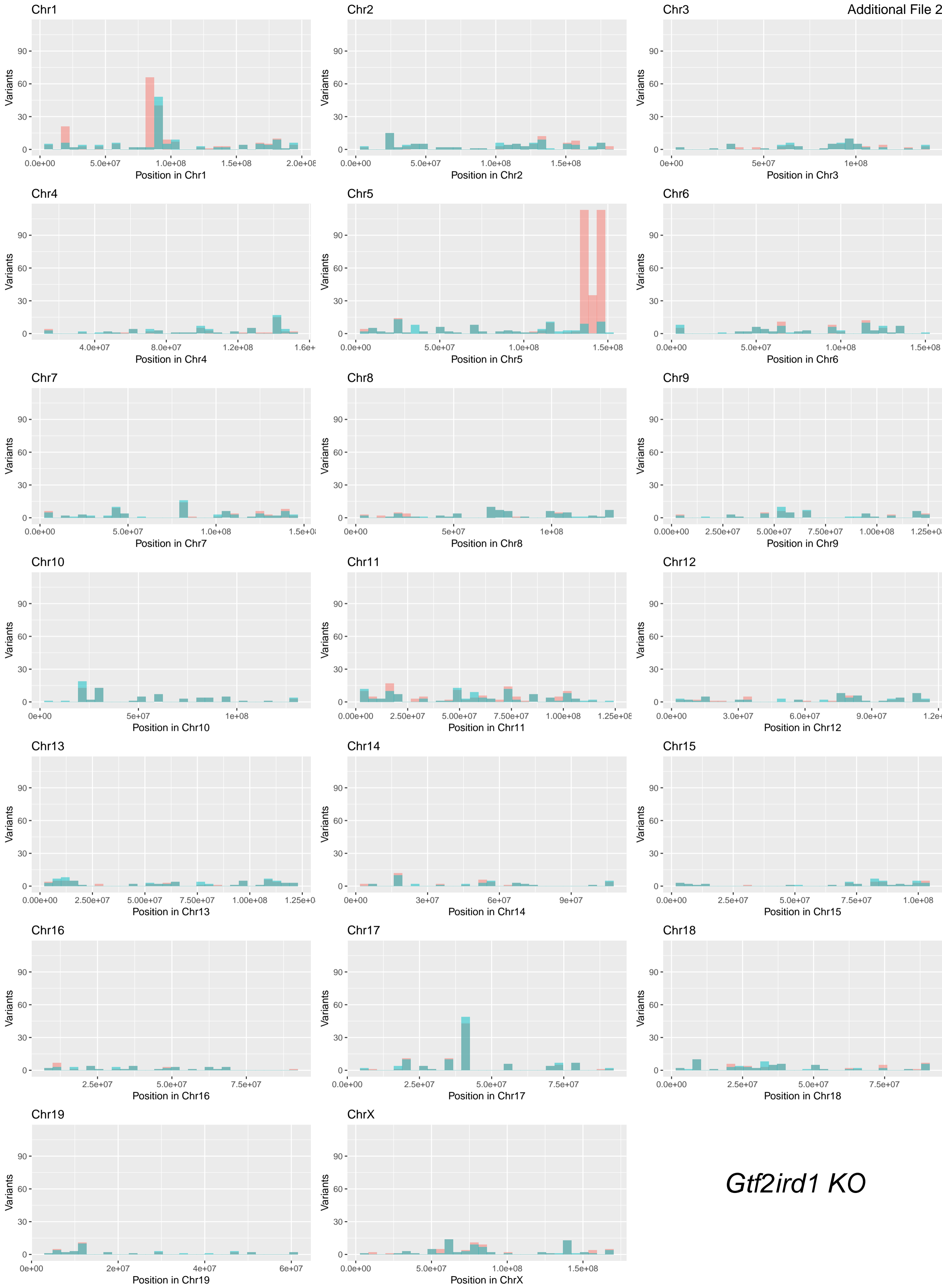*Gtf2ird1* KO

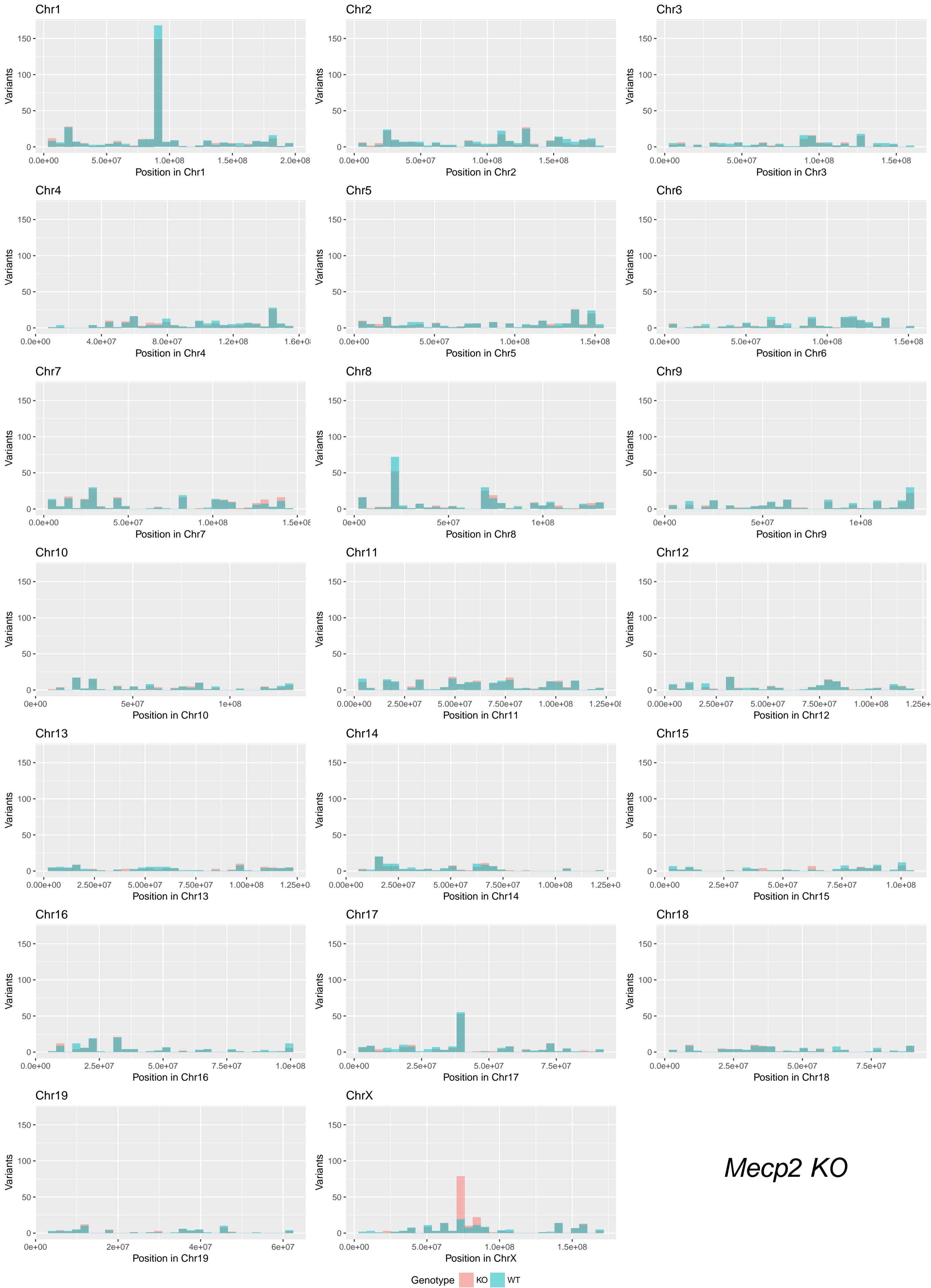

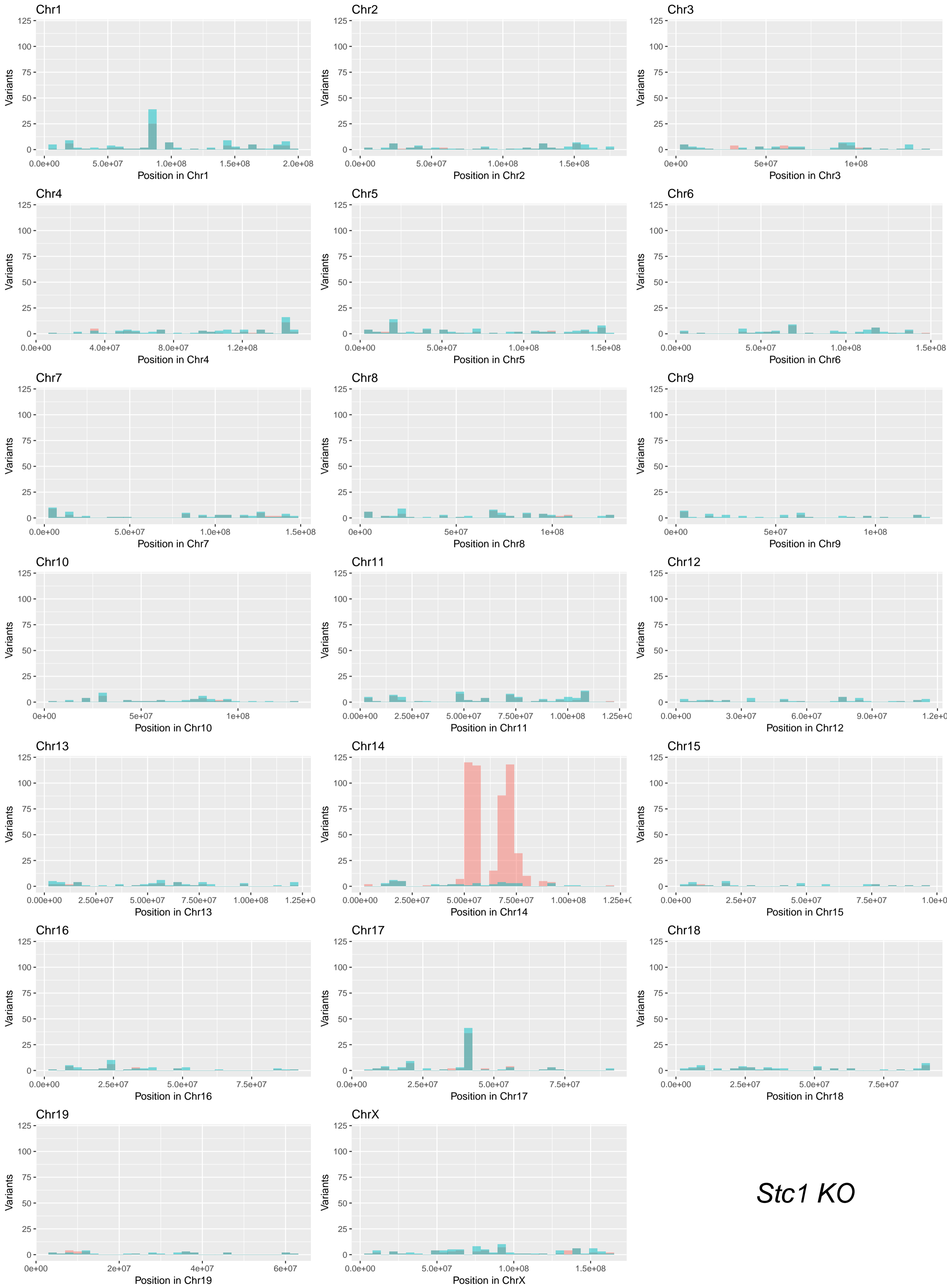

*Stc1* KO

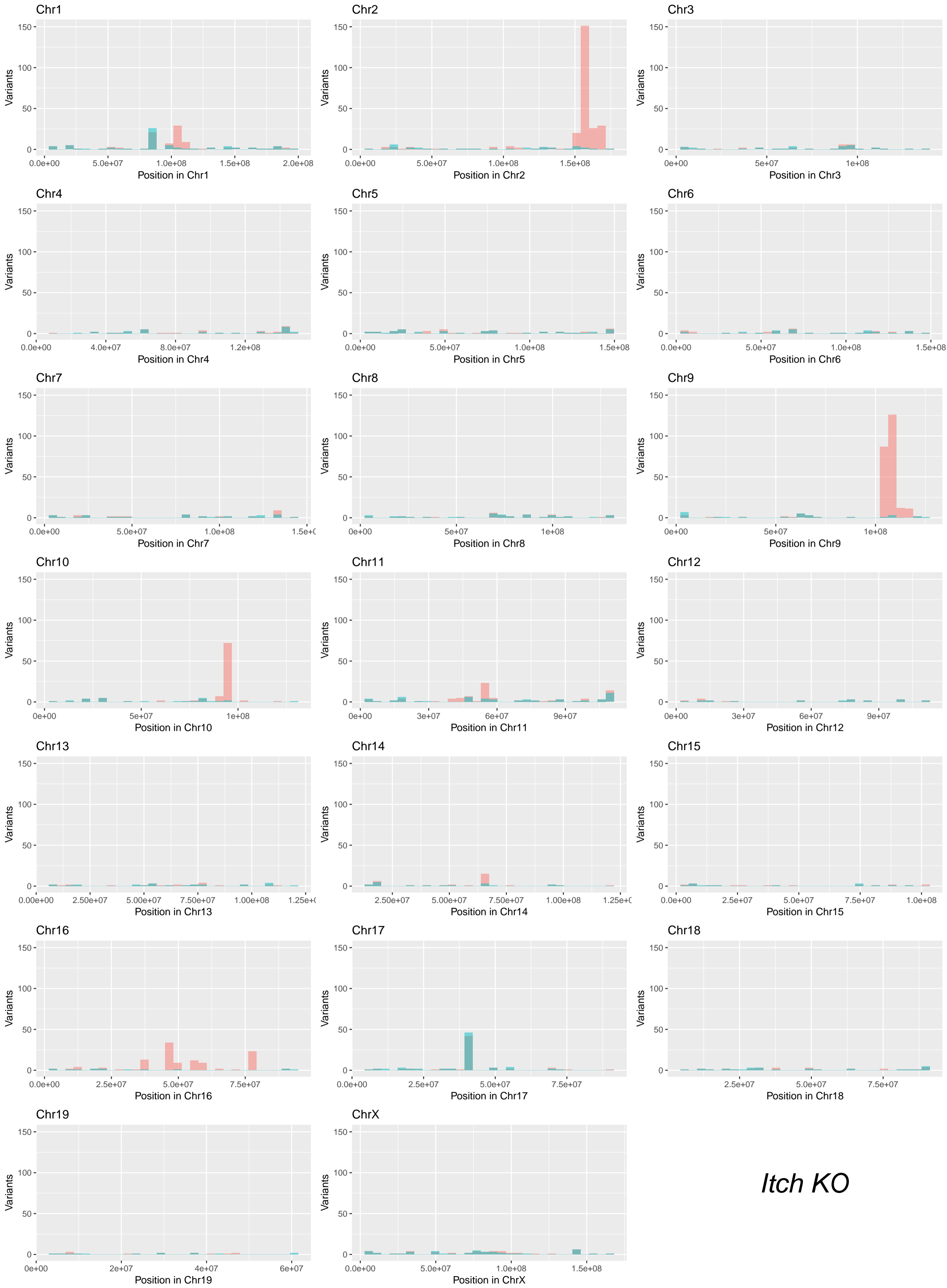

*Itch* KO

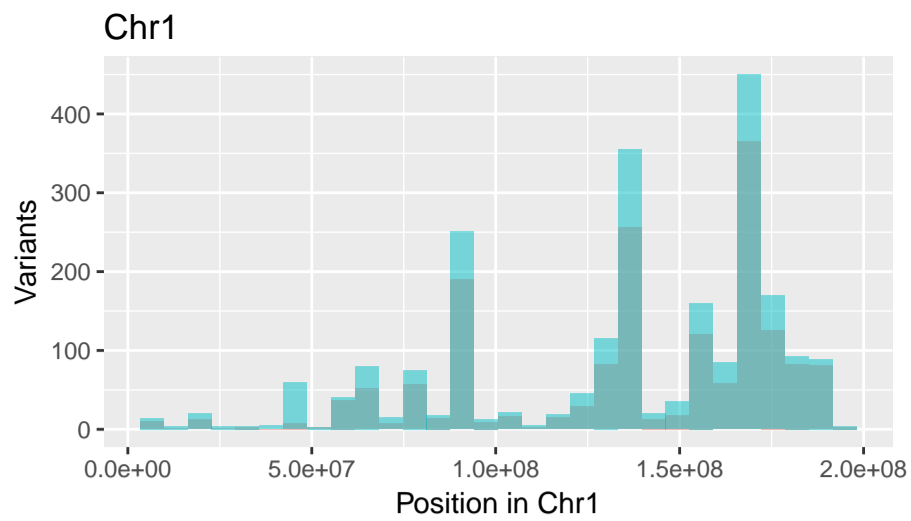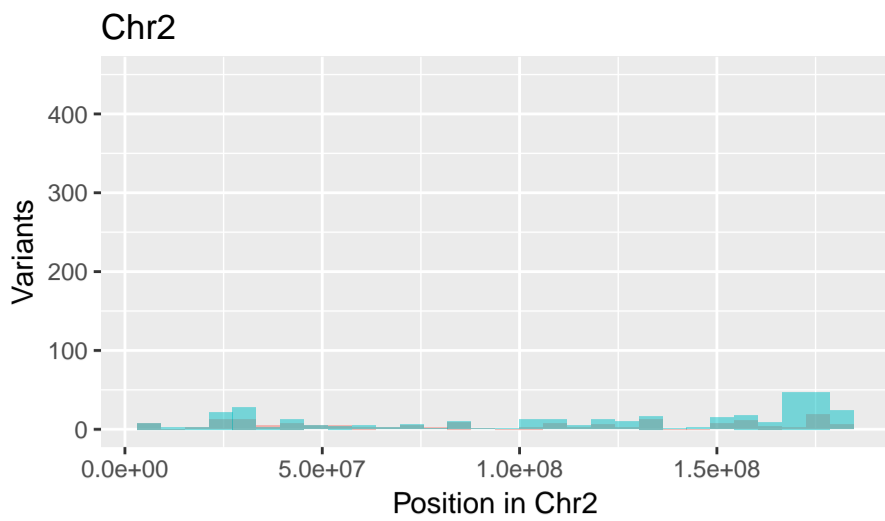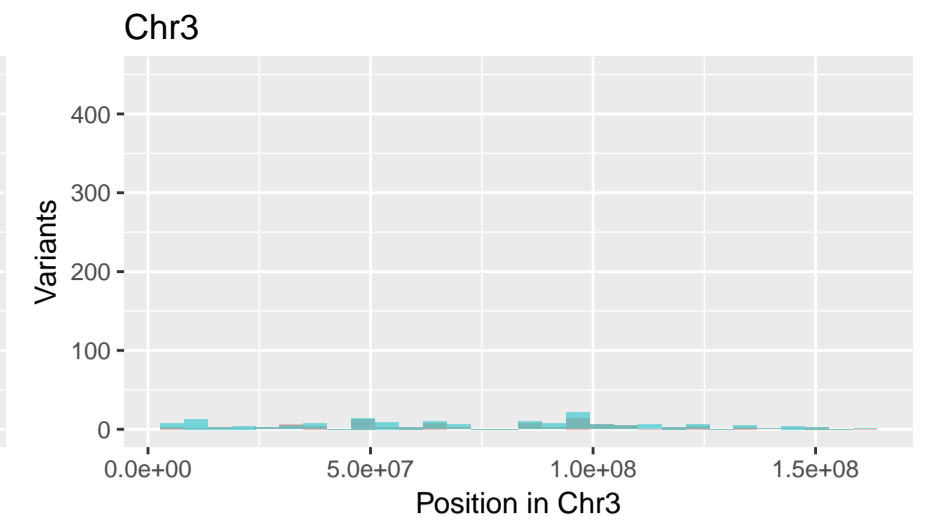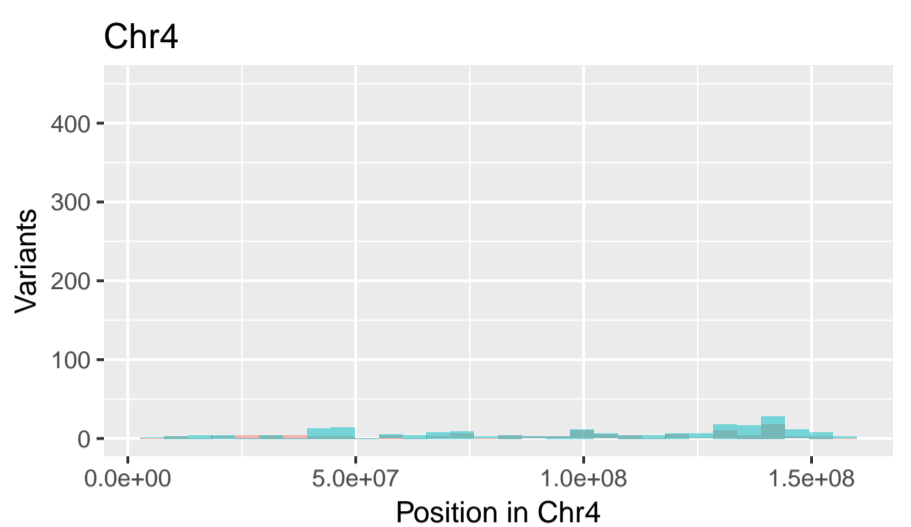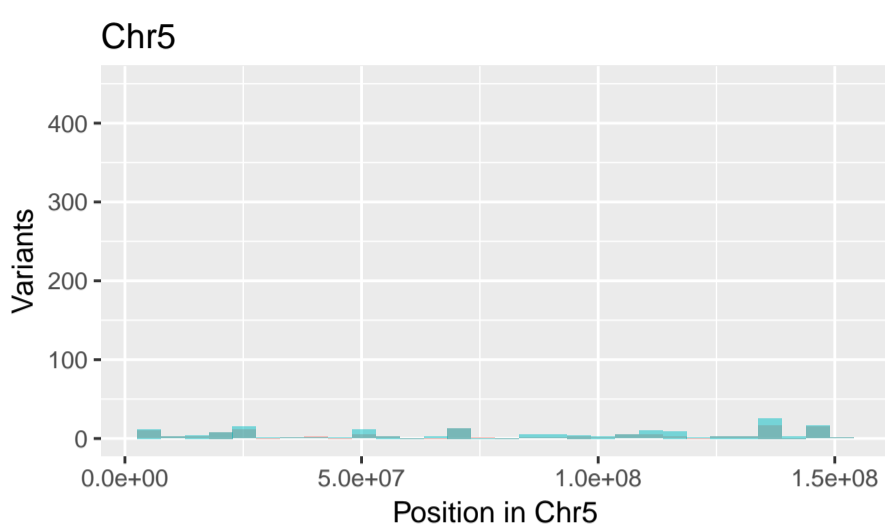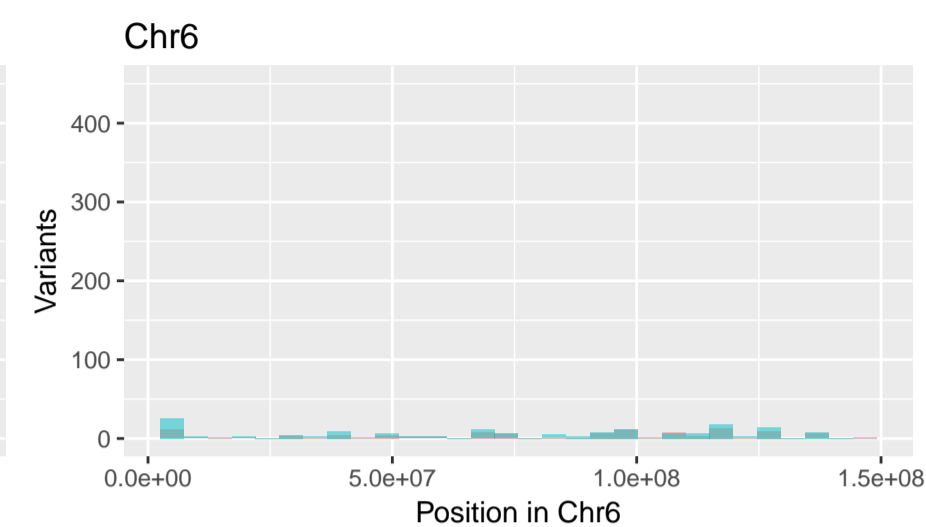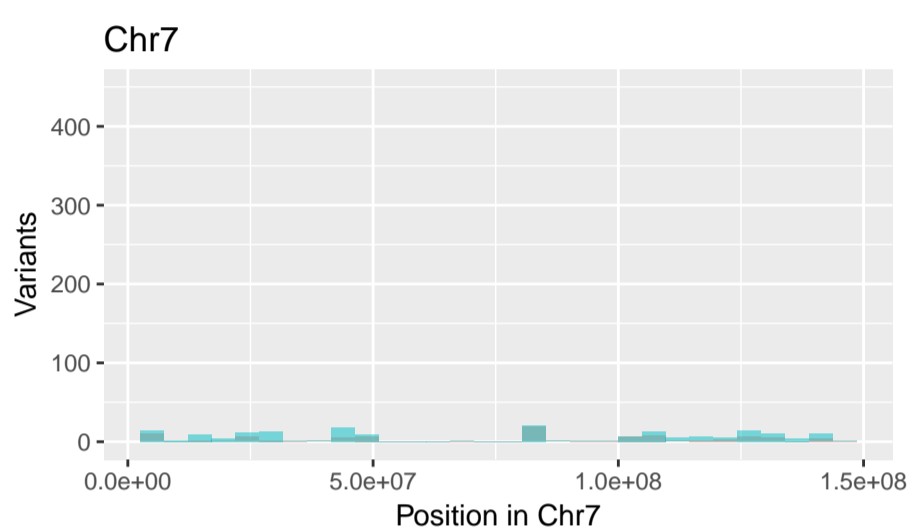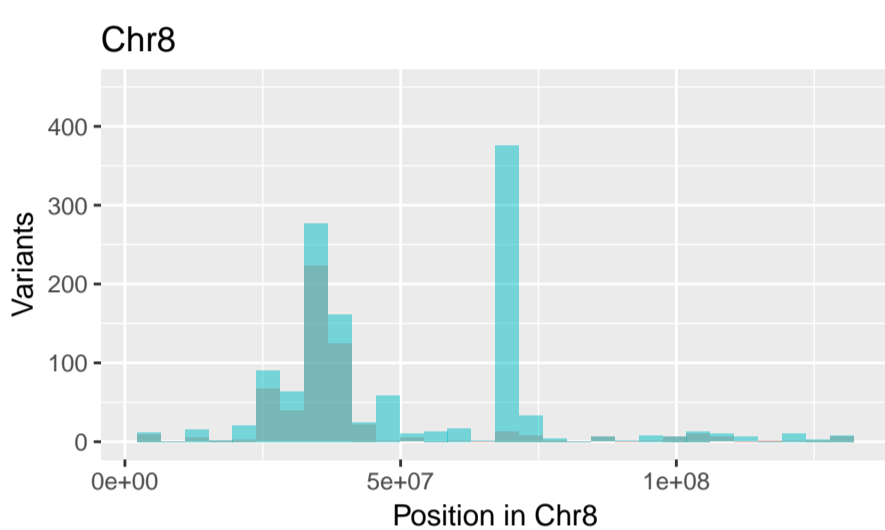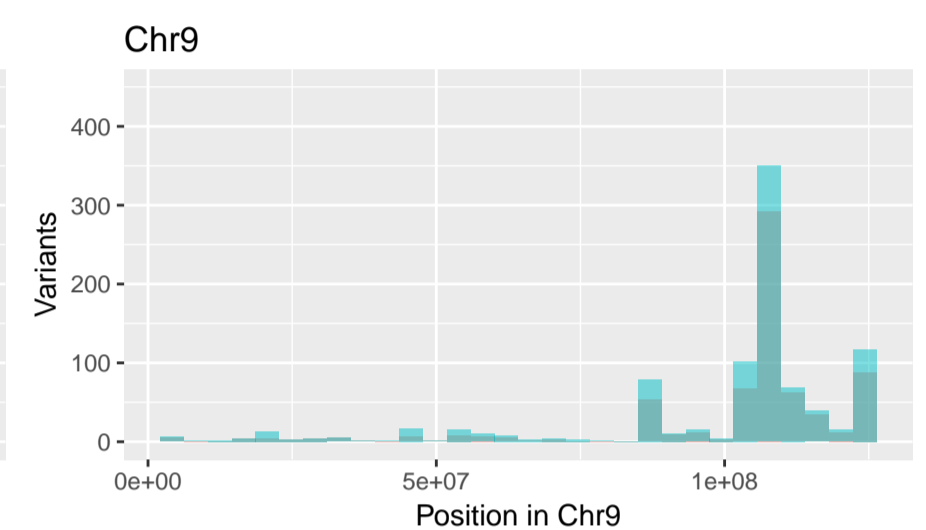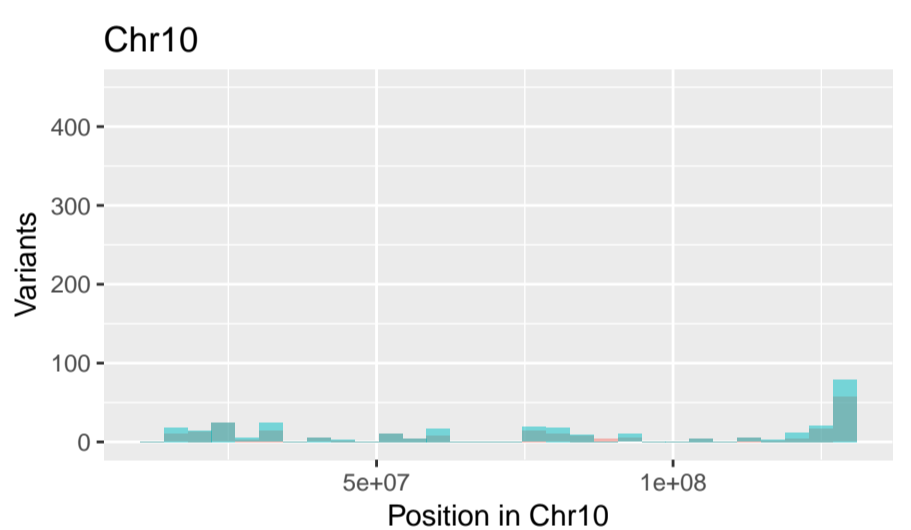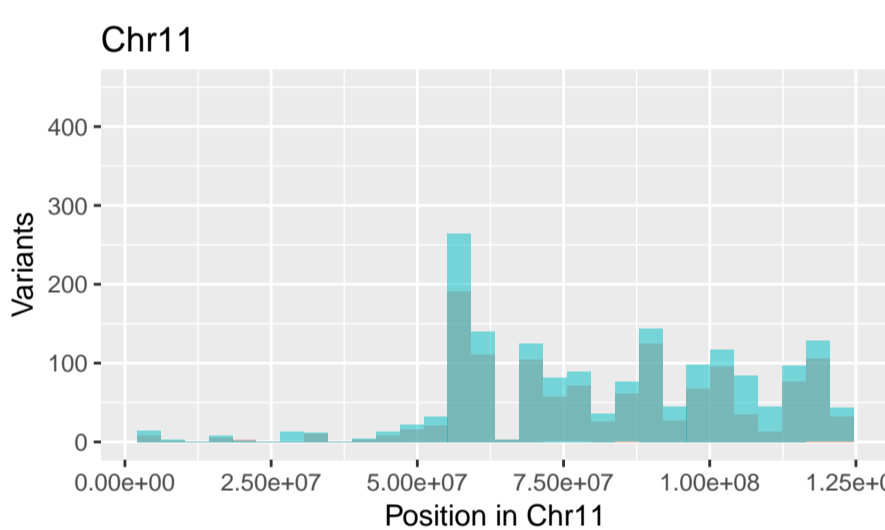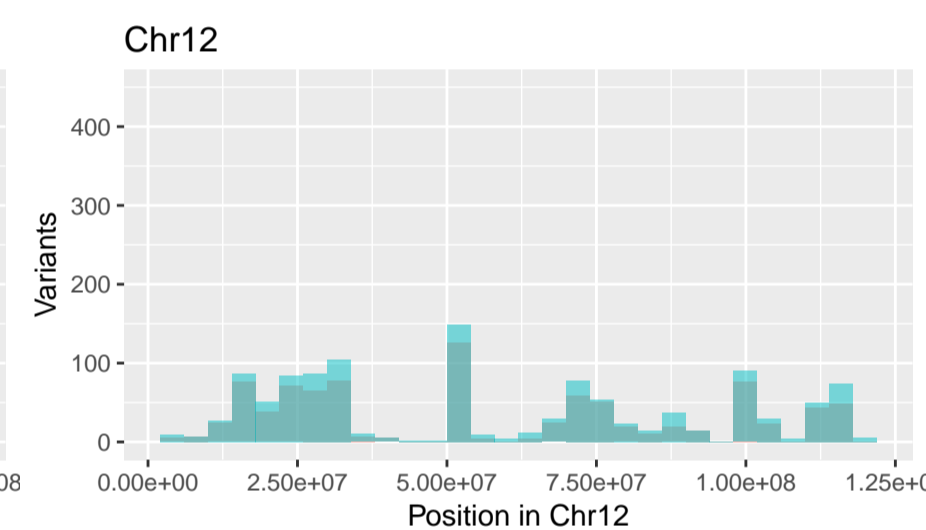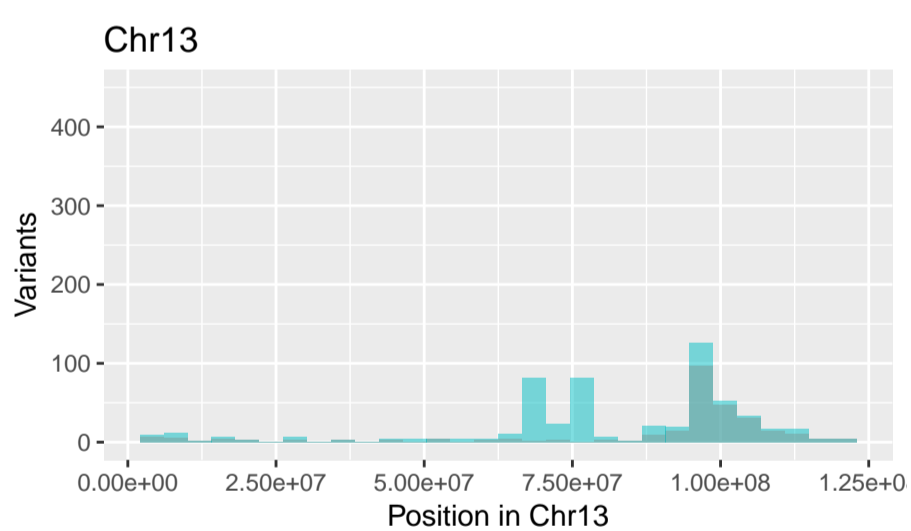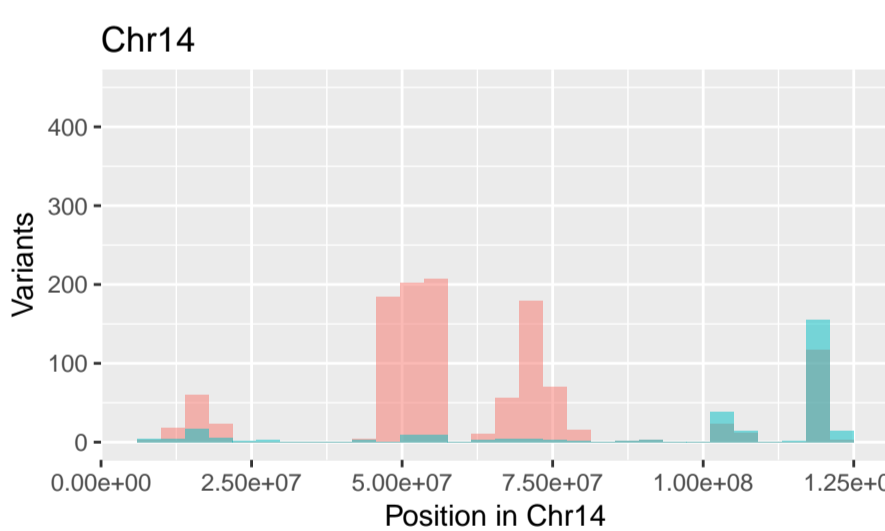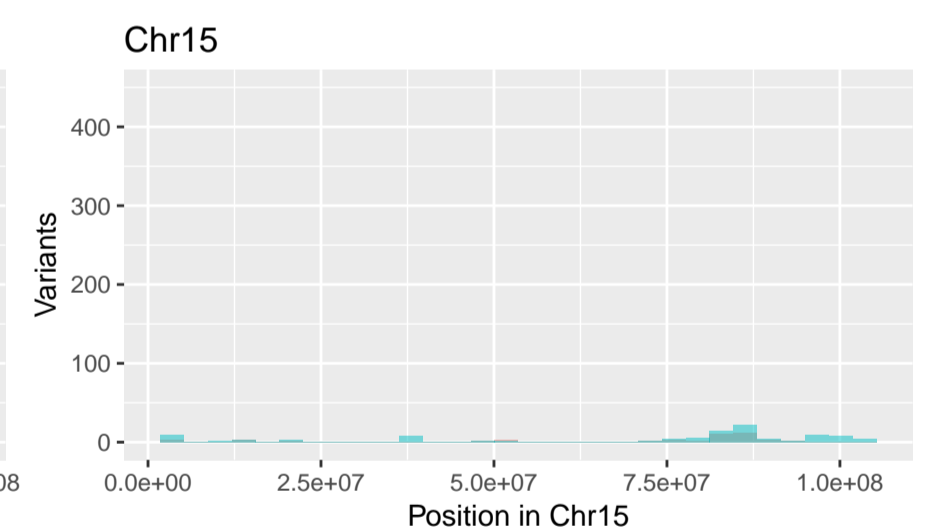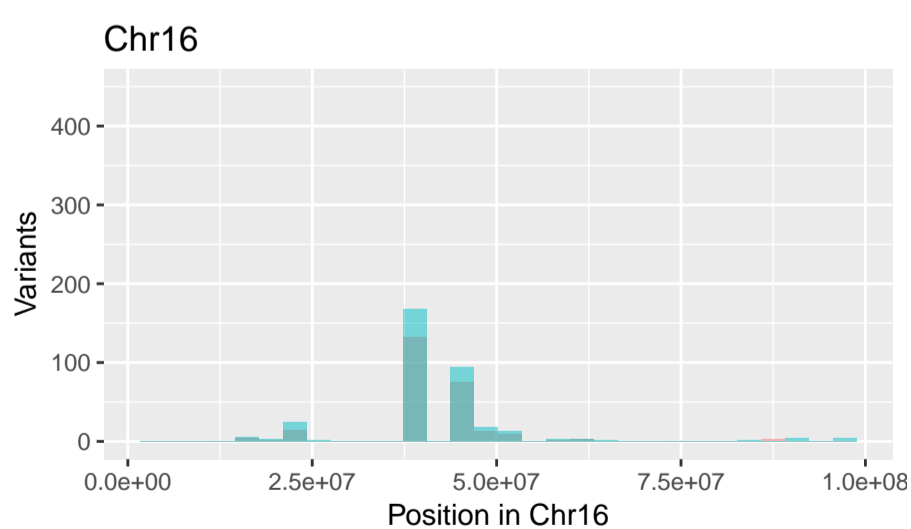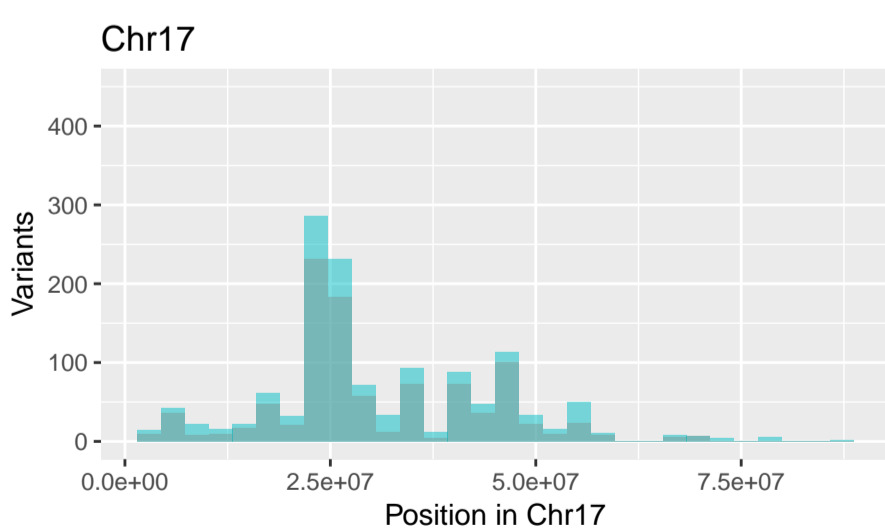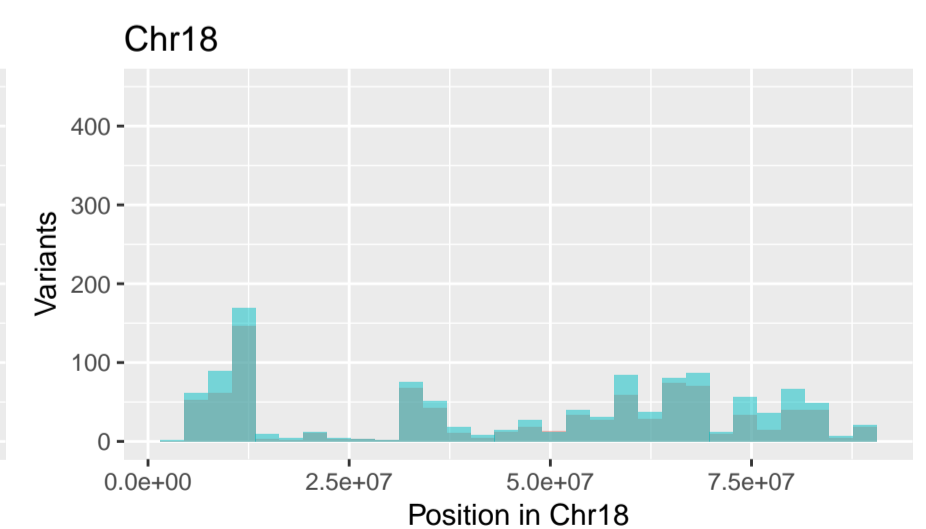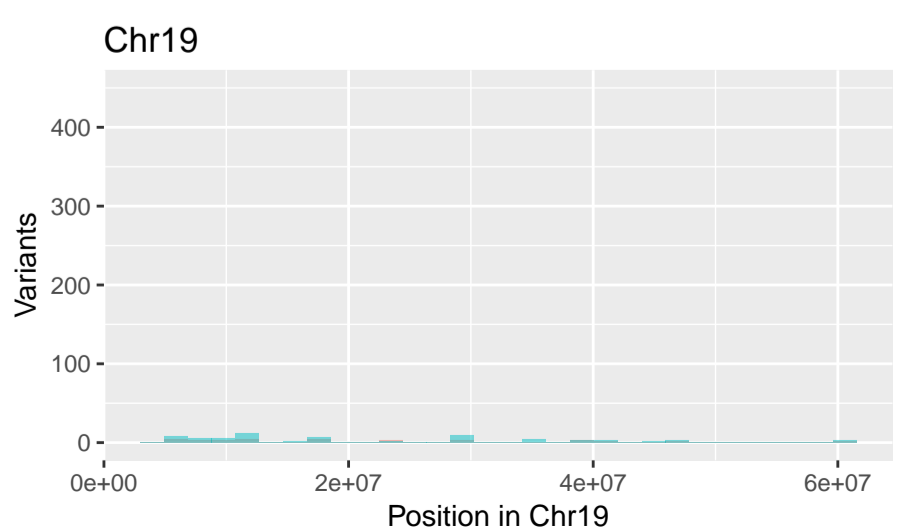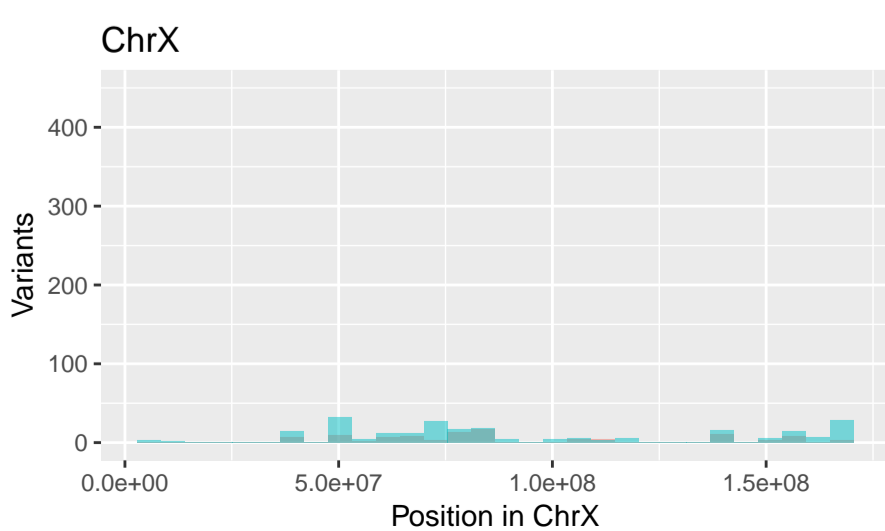

*Sall2* KO

Genotype ■ KO ■ WT

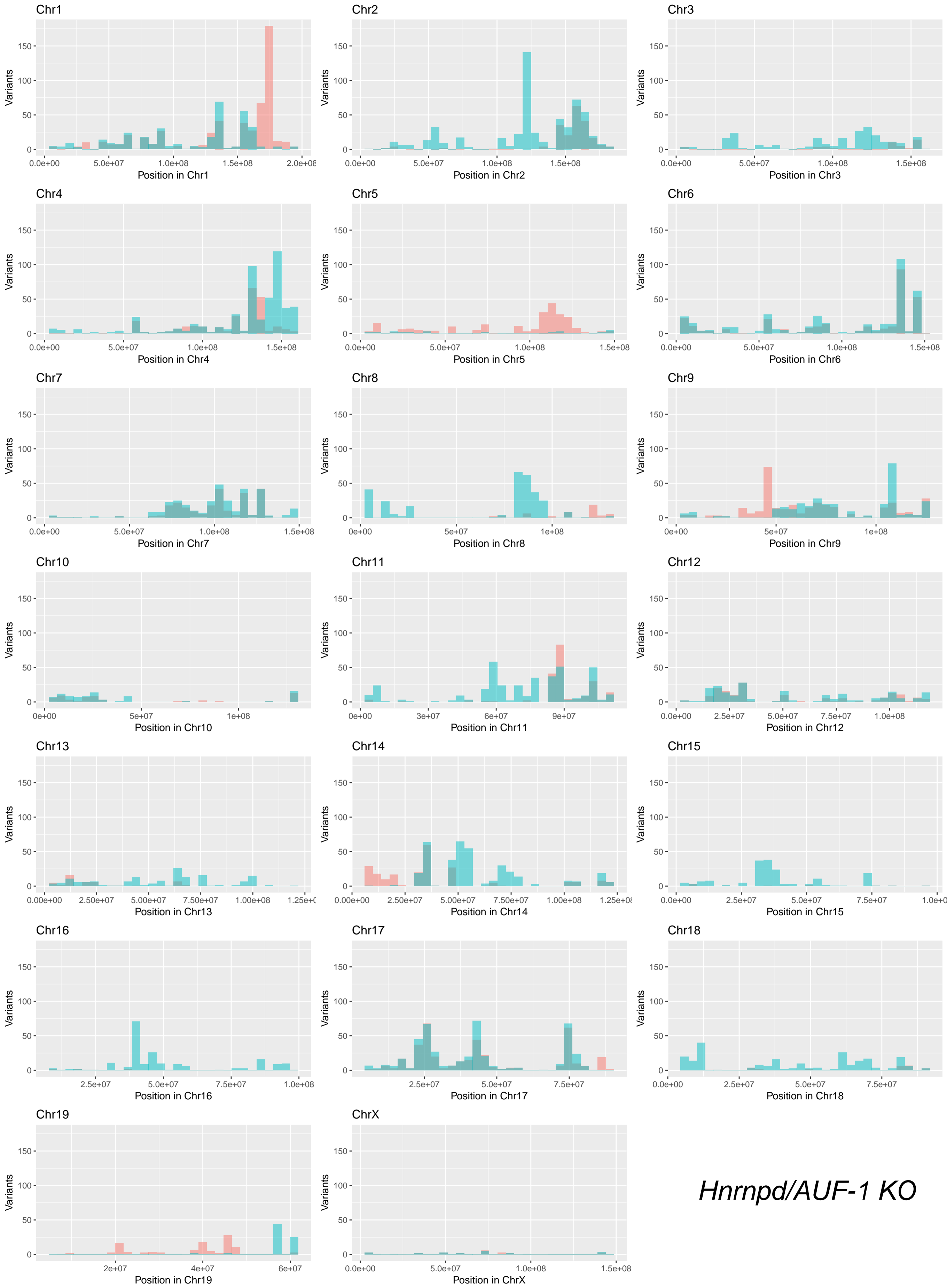

*Hnrnpd/AUF-1 KO*

Supplement: Supplementary file 2 — Whole genome histogram of novel/existing variants in Gtf2ird1, Mecp2, Stc1, Itch, Sall2 and Hnrnpd/AUF-1 KO (RNA-Seq). RNA-Seq samples from Gtf2ird1, Mecp2, Stc1, Itch, Sall2 and Hnrnpd/AUF-1 WT and KO embryos were plotted, including WES samples from GSE115017 (GEO datasets) and E-MTAB-4181 (ArrayExpress). We binned the genomic coordinates of each chromosome every 10 million bases, and plotted the variants of each genotype/condition as frequency histograms according to these positions. In the case of RNA-Seq samples, blue bars represent average variants from WT embryos, and red bars represent the average variants from KO embryos in each case. The biological replicates were as follows: In the Sall2 KO, WT = 1 and KO = 3, in the Itch KO, WT = 2 and KO = 2 and in the four other studies, WT = 3 and KO = 3. (PDF 76 kb) [file 12864_2019_5504_MOESM2_ESM.pdf]

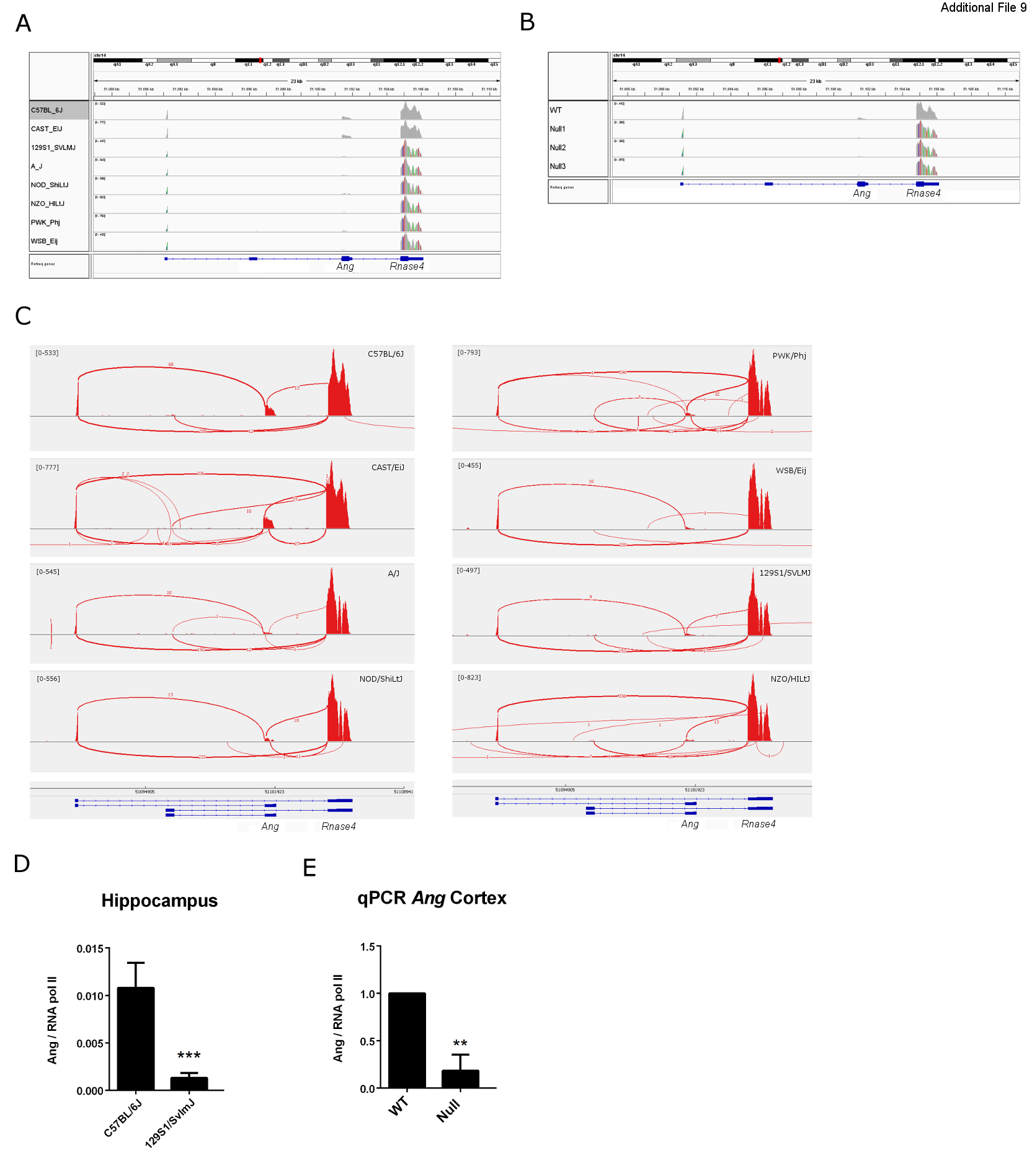

Supplement: Supplementary file 9 — Pervasive downregulation of Ang in 129 mice. A) Left: IGV snapshot of Ang/Rnase4 gene expression across mouse founders (PRJNA228935 accession). C57BL/6J and 129S1/SvImJ strains are placed in the upper panels. The gene model is shown in blue and was obtained from the UCSC server. B) Same snapshots as in (A) across Sall2 RNA-Seq samples. C) Sashimi plots of samples in (A) depicting exon usage as the number of junctions. Per-base expression is plotted on the y-axis of Sashimi plot; genomic coordinates on the x-axis, and the gene structure are represented on the bottom (in blue, obtained from the USCS server). D) Gene counts of Ang from the hippocampus of C57BL/6J and 129S1/SvImJ mice normalized against Polr2a gene counts (GSE76567, N = 6, *** P < 0.001, versus C57BL/6J; Student’s T-test). E) Quantitative real-time PCR of Ang in the cortex coming from Sall2 WT and null mice. RNA from Sall2 WT and null cortex were isolated, reverse transcribed and analyzed by quantitative real-time PCR. Shown are Ang expression levels normalized to Polr2A when compared to levels in WT. (N = 3; data is represented as means ± s.e.m.) *** P < 0.001 versus WT; Student’s T-test. (TIF 382 kb) [file 12864_2019_5504_MOESM9_ESM.tif]

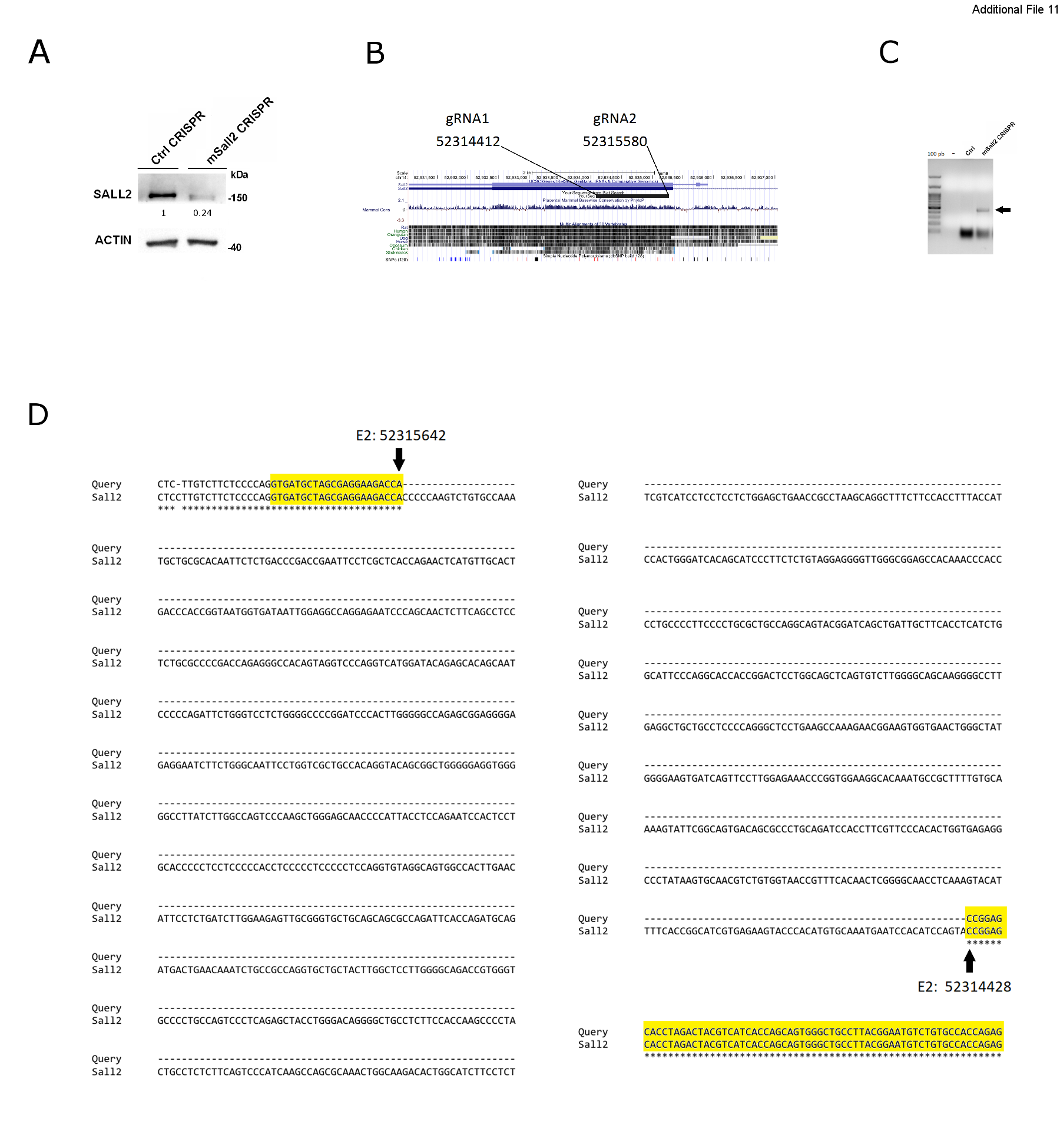

Supplement: Supplementary file 11 — Validation of the murine Sall2 gene deletion by CRISPR-Cas9. A) Representative Western blot for SALL2 and ACTIN in control and Sall2-silenced cells by CRISPR (mSall2 CRISPR) done in Sall2 WT iMEFs. B) We designed a double CRISPR cut to delete a segment of the Sall2 gene. The two CRISPRs (denoted as gRNA one and two) targeted the largest exon of the murine Sall2 gene (exon 2). C). iMEF cells were electroporated with Control CRISPR plasmid or the two mSall2 CRISPR plasmids, and fluorescent cells were enriched by flow-cell cytometry (top 5% of fluorescent cells). We identified the desired deletion from the genomic DNA of a pool of iMEF cells and targeted it with the double CRISPR strategy (amplicon at 500 base pairs in mSall2 lane, denoted with a black arrow). D) Alignment from the Sanger sequencing results of the gel-purified amplicon from (C), depicting the genomic deletion of the Sall2 gene (chromosomal position 52,314,428–52,315,642 on the mm10 build). We highlighted the codifying sequences of the exon two of murine Sall2 gene in yellow. (TIF 808 kb) [file 12864_2019_5504_MOESM11_ESM.tif]
