# Supplementary material for: Streamlined computational pipeline for genetic background characterization of genetically engineered mice based on next generation sequencing data
Source: BMC Genomics. 2019 Feb 12;20:131. doi: 10.1186/s12864-019-5504-9 (PMC6373082; doi:10.1186/s12864-019-5504-9)
Supplement: Supplementary file 3 — Whole genome histogram of novel/existing variants in two WES studies. WES samples from the GEO datasets, GSE115017 and from the SRA archive E-MTAB-4181, were plotted as in Additional file 2. The samples selected from the first study were GSM3163042 (C57BL/6J) with GSM3163051 (C57BL/6J mixed with DBA2) and SAMEA3940161 (Tumor1) with SAMEA3940166 (Tumor6) for the second study. A Cochran-Armitage test was included after every plot. (PDF 38 kb) [file 12864_2019_5504_MOESM3_ESM.pdf]

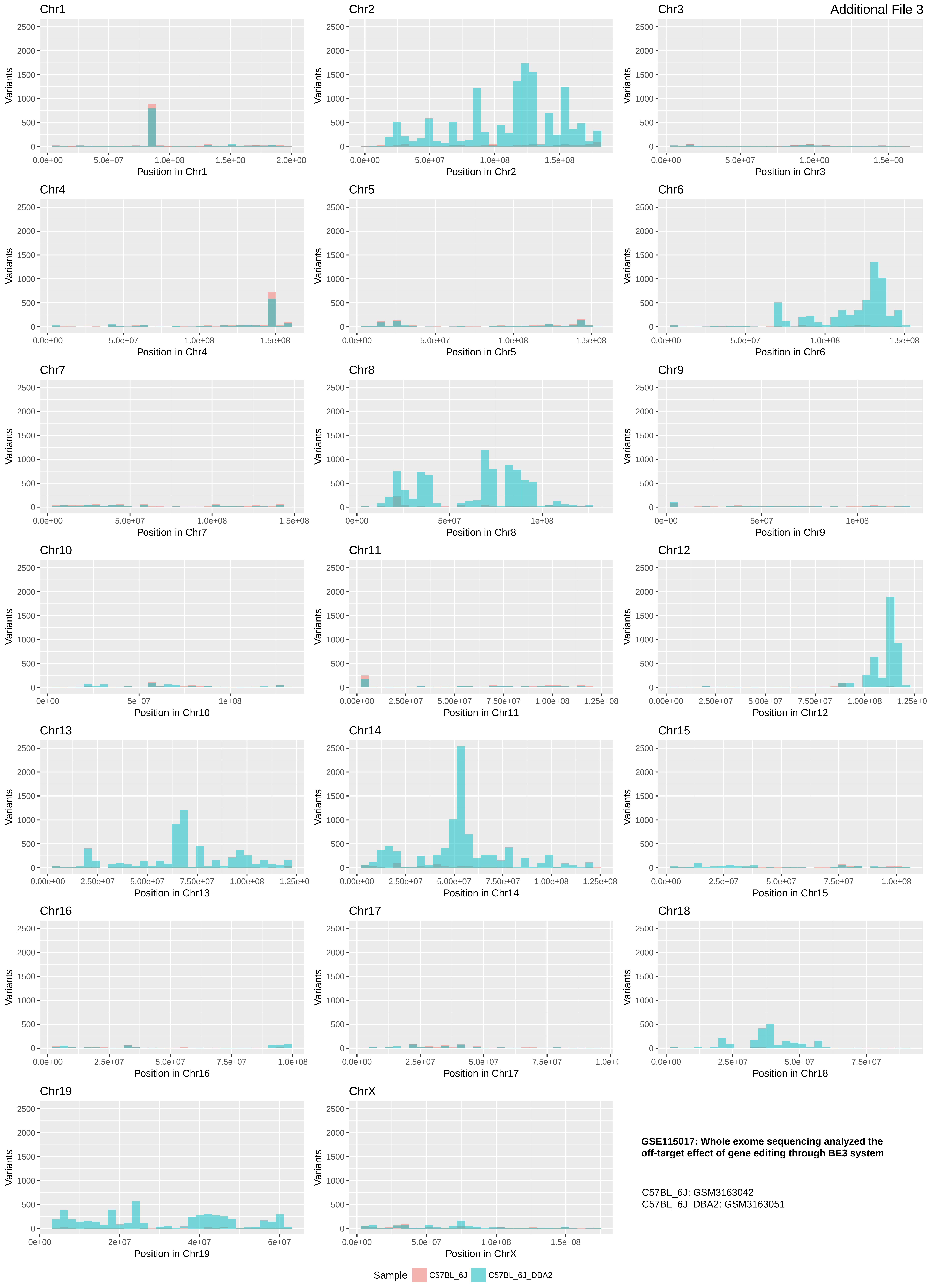

GSE115017: Whole exome sequencing analyzed the off-target effect of gene editing through BE3 system

C57BL_6J: GSM3163042
C57BL_6J_DBA2: GSM3163051

Cochran-Armitage Test for GSE115017 study

	Chromosome	p.value	significance
1	chr1	0.703968687262022	FALSE
2	chr2	0.0376252488642597	TRUE
3	chr3	0.0015707971661551	TRUE
4	chr4	0.424806621902029	FALSE
5	chr5	0.636836935900358	FALSE
6	chr6	4.62047387612157e−139	TRUE
7	chr7	0.802700020992426	FALSE
8	chr8	8.95380644203593e−09	TRUE
9	chr9	0.00735541294217049	TRUE
10	chr10	5.10305372706502e−09	TRUE
11	chr11	0.605198595777314	FALSE
12	chr12	1.81272519265802e−305	TRUE
13	chr13	2.59050279538819e−05	TRUE
14	chr14	1.58901133875399e−38	TRUE
15	chr15	4.74700984937771e−40	TRUE
16	chr16	3.12136895806258e−16	TRUE
17	chr17	0.136212104650778	FALSE
18	chr18	0.000986824546301305	TRUE
19	chr19	0.600252871658779	FALSE
20	chrX	0.111456362939433	FALSE

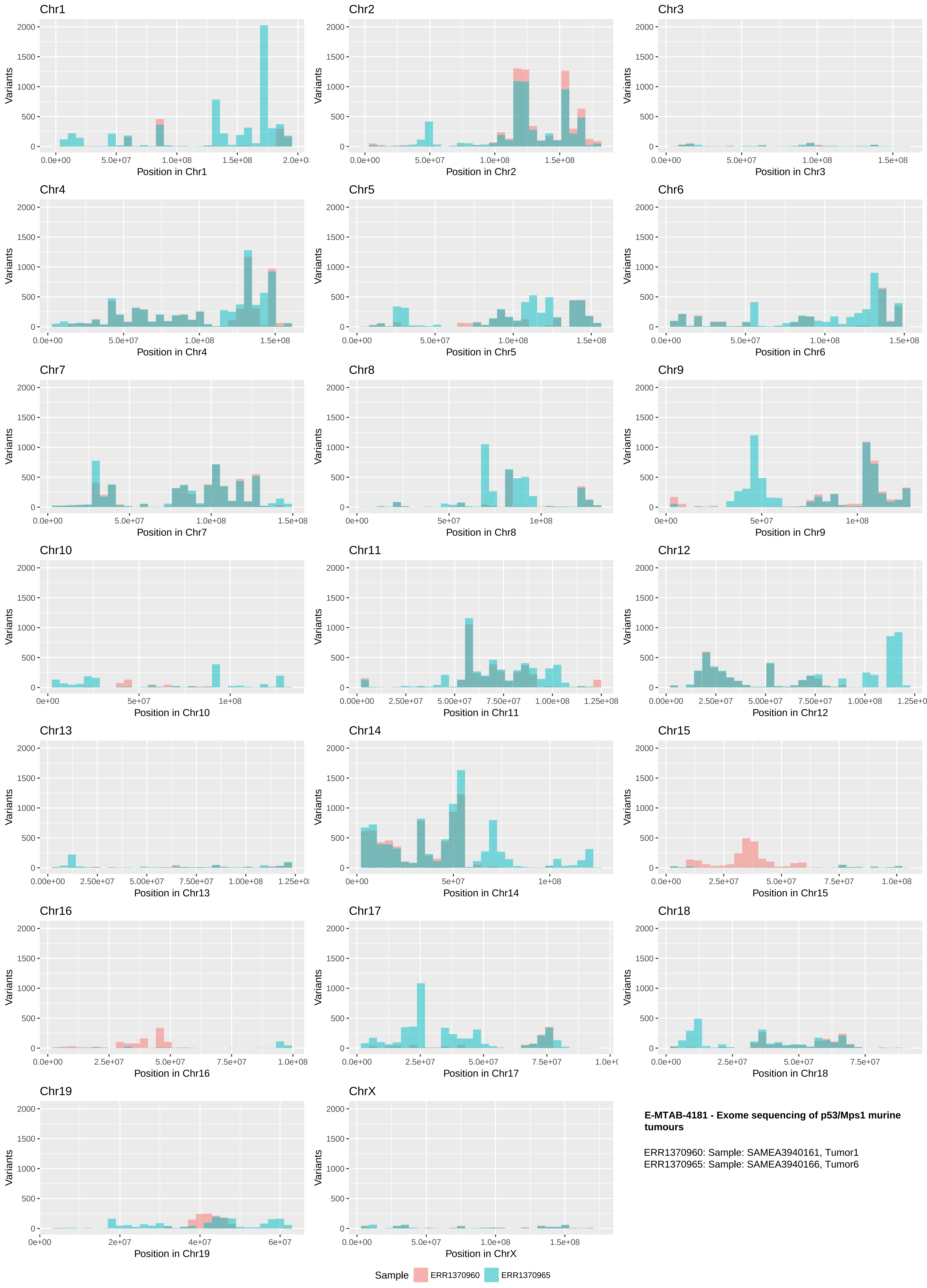

E-MTAB-4181 - Exome sequencing of p53/Mps1 murine tumours

ERR1370960: Sample: SAMEA3940161, Tumor1
ERR1370965: Sample: SAMEA3940166, Tumor6

Cochran-Armitage Test for E-MTAB-4181 study

	Chromosome	p.value	significance
1	chr1	2.9030338271892e−21	TRUE
2	chr2	3.0012892403328e−71	TRUE
3	chr3	0.876379289027447	FALSE
4	chr4	0.000731675844692207	TRUE
5	chr5	5.51660846537133e−17	TRUE
6	chr6	7.56596812084302e−20	TRUE
7	chr7	0.000148505156686152	TRUE
8	chr8	2.08021715113292e−28	TRUE
9	chr9	4.11958717751639e−192	TRUE
10	chr10	0.0256874424850623	TRUE
11	chr11	2.87969776356073e−12	TRUE
12	chr12	2.67786940875146e−295	TRUE
13	chr13	5.11414834033401e−19	TRUE
14	chr14	1.33595760663085e−190	TRUE
15	chr15	4.86567876395169e−69	TRUE
16	chr16	3.64096548716859e−94	TRUE
17	chr17	4.21103051353847e−175	TRUE
18	chr18	2.8203304812456e−109	TRUE
19	chr19	0.351409090137602	FALSE
20	chrX	0.000175492277961431	TRUE
